# Prescribing Experiences, Potentials, and Challenges of Digital Health Applications in the Field of Hormones and Metabolism: Cross-Sectional Survey Study of Health Care Providers in Germany

**DOI:** 10.2196/77792

**Published:** 2025-12-31

**Authors:** Melanie Mäder, Dirk Müller-Wieland, Tobias Wiesner, Tonio Schoenfelder, Carsta Militzer-Horstmann, Ria Heinrich, Mareike Geisler, Dennis Häckl

**Affiliations:** 1 Chair for Health Economics and Management Faculty of Economics and Management Science Leipzig University Leipzig Germany; 2 Scientific Institute for Health Economics and Health System Research Leipzig Germany; 3 Department of Medicine I Universitätsklinikum Aachen Aachen Germany; 4 Medical Care Center for Metabolic Medicine Leipzig Germany

**Keywords:** chronic disease management, diabetes mellitus type 2, digital health applications, health care providers, medical decision-making, obesity, overweight

## Abstract

**Background:**

In 2020, the global prevalence of overweight and obesity was approximately 42%. One of the most common associated conditions is type 2 diabetes mellitus, which had a global prevalence of around 10.5% in 2021. Digital health applications (DiHA), which can be prescribed as certified medical devices in Germany, have been shown to effectively support disease management in patients with overweight and diabetes mellitus. However, little is known about DiHA-prescribing behavior of health care providers (HCPs) specializing in hormones and metabolism or about potential barriers to prescribing these applications.

**Objective:**

This study aimed to assess HCPs’ experience with and willingness to prescribe DiHA in the field of hormones and metabolism. In addition, it sought to examine the patient-relevant health care effects that HCPs perceive as potentially achievable or have already observed with DiHA use, as well as the barriers they perceive to prescribing these applications.

**Methods:**

An online questionnaire was developed based on preliminary studies and a literature review consisting of 86 items covering 6 key areas: experience and willingness to prescribe, health care effects, barriers, scientific evidence, digital affinity, and sociodemographics. The anonymous survey was distributed via the German Diabetes Association to 6035 HCPs in Germany between August 2 and October 9, 2024. Descriptive analyses, as well as correlation and regression analyses, were conducted.

**Results:**

A total of 350 HCPs participated in the survey (response rate=5.8%). Although the low response rate may limit generalizability, the findings provide insights into prescribing behavior within this specialty. More than half (187/350, 53.4%) had never prescribed any of the 54 DiHA available at the time of the survey, with 47.6% (89/187) citing a lack of experience as the primary reason. Among those who had prescribed a DiHA (163/350, 46.6%), the majority (139/163, 85.3%) had prescribed 1 of the 8 DiHA available for obesity or diabetes mellitus. Looking ahead, 42.9% (149/348) of all surveyed HCPs stated that they were either very unlikely (83/348, 23.9%) or somewhat unlikely (66/348, 19%) to prescribe these DiHA in the next 12 months. The greatest perceived benefits of DiHA were improvements in self-management, health literacy, and adherence. The main barriers to prescribing DiHA in the field of hormones and metabolism included inadequate reimbursement for ancillary medical services, poor compatibility with existing practice software, and a lack of digital affinity or motivation among patients.

**Conclusions:**

DiHA have not yet been fully integrated into standard health care. To improve prescribing, we recommend integrating DiHA into medical guidelines, ensuring proper reimbursement, and involving HCPs in the pricing and health-economic evaluation of DiHA. The recommendations outlined should be considered to maximize DiHA’s potential and improve HCPs' acceptance, providing valuable insights for health policy to enhance the integration, reimbursement, and use of DiHA.

## Introduction

Digital interventions provide substantial opportunities for enhancing the management of chronic conditions [1], particularly by strengthening patient empowerment, supporting self-management [2], and improving treatment adherence [3]. They are especially relevant in the context of chronic diseases such as overweight, obesity, and type 2 diabetes mellitus (T2DM), which are associated with a significant clinical and health-economic burden [4,5].

In 2020, approximately 42% of the global population was classified as overweight (n=1.39 billion) or obese (n=0.81 billion), and this proportion is projected to rise to 54% by 2035, representing an estimated 1.77 billion individuals with overweight and 1.53 billion individuals with obesity [5]. T2DM is among the most prevalent secondary conditions associated with obesity, with a global prevalence of approximately 10.5% in 2021, projected to rise to 12.2% by 2045 [4]. Both obesity and diabetes mellitus (DM) are associated with significant impairments in quality of life (QoL) [6-8].

Digital interventions have the potential to enhance clinical outcomes and improve QoL in individuals with obesity or DM [9]. In DM applications, this is primarily reflected in clinically significant reductions in glycated hemoglobin (HbA_1c_) levels [10,11], while in obesity applications, it is demonstrated by clinically significant reductions in body weight [12,13]. Digital health applications (DiHA) were introduced into the German health care system in 2019 as components of standard care covered by statutory health insurance (SHI) funds. Applications are eligible for DiHA status if they successfully complete the evaluation procedure conducted by the Federal Institute for Drugs and Medical Devices (Bundesinstitut für Arzneimittel und Medizinprodukte [BfArM]) and demonstrate a measurable benefit compared with standard therapy. As of December 7, 2025, the DiHA directory comprises 57 registered applications. Following a confirmed diagnosis (*ICD-10* [*International Statistical Classification of Diseases, Tenth Revision*] code), these applications can be prescribed by physicians or psychotherapists, underscoring the essential role of these professional groups in the deployment of DiHA and the advancement of a digitally enabled health care system.

Digital interventions like DiHA, and their acceptance and use by health care providers (HCPs), have been extensively investigated through quantitative surveys in Germany which indicate that HCP’s willingness to prescribe them remains low. A nationwide survey in 2020 found that 37% of general practitioners (GPs; N=51) reported a rather high or high willingness to prescribe DiHA [14]. Another study conducted between December 2020 and January 2021 (N=1308) revealed that only 30.3% of HCPs planned to continue prescribing DiHA in the future, despite acknowledging their benefits [15]. A 2022 survey among GPs (N=3829) in 4 German states found an even lower willingness to prescribe at just 13% [16]. In contrast, a nationwide study of diabetes specialists (N=538) conducted between September 2021 and April 2022 showed that 51% perceived health apps as potentially beneficial for T2DM, though further research was needed to evaluate their strengths and weaknesses [17]. A more recent survey in 2023 reported that 38.5% of GPs (N=97) in Giessen were very likely to prescribe DiHA in the next 12 months, highlighting the ongoing need for education and information to improve acceptance [18]. The studies mentioned indicate significant room for improvement in prescribing willingness, but all these studies either focus on DiHA use and acceptance by GPs in a broad context, without addressing specific indication areas, or they examine digital interventions in general, without specifically focusing on DiHA. Focusing the survey on a specific indication area—hormones and metabolism in this case—is crucial to ensure the validity and relevance of the results. DiHA vary widely in terms of application profiles, levels of evidence, acceptance, and clinical relevance across different medical specialties. A generalized perspective across all indications would obscure these differences and prevent meaningful conclusions from being drawn regarding the use, evaluation, or implementation of DiHA within a specific health care context. Therefore, to better understand indication-specific prescribing experiences, potential benefits, and challenges associated with DiHA, it is necessary to analyze DiHA adoption within individual medical specialties. So, the planned research project focuses on prescribing experiences, potential benefits, and challenges associated with DiHA within the field of hormones and metabolism—hereinafter referred to as “hormones and metabolism digital health applications” (HM-DiHA)—and aims to address the following research questions:

Our primary questions are as follows:

1. What is the experience and willingness of HCPs to prescribe HM-DiHA?
2. What patient-relevant health care effects can potentially be achieved and have already been achieved using HM-DiHA?
3. What barriers do HCPs perceive regarding the prescription of HM-DiHA?
4. What actionable recommendations can be derived from the findings to enhance HCPs’ acceptance of HM-DiHA?

Our secondary question is as follows:

Is there a correlation between HCPs’ (1) previous experience with DiHA prescription, (2) prescription frequency, and (3) intention to prescribe HM-DiHA and the following characteristics: gender, age, medical specialization, activity within the framework of SHI care, additional professional qualification (eg, diabetologist, adiposiologist, etc) professional experience, location of practice (federal state and practice environment), working model, number of patients treated per quarter, and digital affinity?

## Methods

### Study Design

To address the predefined research questions, the authors used a cross-sectional study design to gather comprehensive opinions and experiences from HCPs in the field of hormones and metabolism; the Ethics Advisory Board of Leipzig University reviewed the application for the research project using a simplified procedure and concluded that there were no ethical objections to its implementation. In a rapidly evolving digital health care landscape, this design offers valuable insights into the current levels of acceptance and implementation of HM-DiHA in clinical practice. With the support of the German Diabetes Association (GDA), a nationwide quantitative online survey was conducted in Germany between August and October 2024. The GDA is a medical-scientific professional society with approximately 9300 members, including physicians in clinics and practices, scientists, psychologists, pharmacists, and other specialists in the field of diabetology [19]. Recruiting participants through the GDA proved to be effective, as its members possess a high level of expertise in managing chronic conditions such as obesity or DM—an area in which DiHA are particularly relevant. Targeting this specialized group ensured both the thematic relevance of the survey and a practice-oriented assessment of DiHA usage and acceptance.

### Survey Instrument

The questionnaire was designed based on preliminary studies by the authors and an extensive literature search on the individual key topics: (1) experiences and willingness to prescribe (14 items) [14-16], (2) health care effects (potential and actual experience; 17 items each) [20], (3) barriers (11 items) [21], (4) study evidence (7 items) [22], (5) digital affinity (4 items), and (6) sociodemographics (16 items). The questionnaire thus comprised a total of 86 items. The number of items answered varied depending on prior responses (eg, blocks were skipped if no DiHA had been prescribed).

Ordinal scales were predominantly used to operationalize individual theme blocks (especially 2-4) to allow the HCPs to answer intuitively while ensuring a sufficient level of information and the highest data quality possible. These ordinal scales were unipolar rating scales, designed to represent the graduated characteristics of individual items. All scale markers were expressed through verbal labels using linguistically clear and widely recognized formulations [23] to facilitate quick responses. A 5-point rating scale was deliberately chosen, as scales with 5 to 7 levels have proven effective and offer optimal psychometric properties [23]. The use of an odd-numbered scale ensured the inclusion of a neutral middle category to capture potential uncertainties in assessments. Additionally, participants had the option to select “I don’t know,” which explicitly indicated difficulties in making an assessment. In some cases, nominal and interval scales were also applied. Alongside standardized questions, open-ended questions were included (eg, regarding problems and barriers to prescribing). The full translated questionnaire is available in Multimedia Appendix 1. The questionnaire was refined through multistage pretesting, including expert reviews and a pilot with 5 HCPs under field conditions; however, no formal psychometric validation (eg, assessment of internal consistency or reliability testing) of the instrument was conducted.

### Recruitment and Sample

Between August 2 and October 9, 2024, the GDA invited 6035 HCPs via email, sent through a mailing list, to participate in an anonymous online survey, with a reminder sent on September 26. The survey targeted all GDA-registered HCPs in the field of hormones and metabolism eligible to prescribe DiHA, representing a full census of this group.

### Data Analysis

Data analysis was performed using SPSS software (version 23; IBM Corp). We presented data using descriptive statistical methods such as frequency, cross-tables and bar charts, as well as individual characteristic values. These included measures of central tendency, such as the mean and median, and measures of dispersion, such as SD, IQR, and range [23].

Bivariate correlation analysis was used to assess both the strength (weak vs strong) and, where applicable, the direction (positive vs negative) of correlations. Depending on the scale level of the variables under investigation, we applied different correlation coefficients (*r*):

- Nominal × Nominal or Nominal × Ordinal (Cramér V): 0.1≤V≤0.29 small effect size; 0.3≤V≤0.49 medium effect size; V≥0.5 large effect size [24]
- Nominal × Metric (eta coefficient, η; 0=no correlation, 1=perfect correlation): 0.100≤η≤0.242 small effect size; 0.243≤η≤0.370 medium effect size; η≥0.371 large effect size [24]
- Ordinal × Ordinal or Ordinal × Metric (Spearman rank correlation coefficient, ρ; –1=perfect negative correlation, 0=no correlation, +1=perfect positive correlation): 0.1≤ρ≤0.29 small effect size; 0.3≤ρ≤0.49 medium effect size; ρ≥0.5 large effect size [24]

The significance level (α) was set at 5% (ie, α=.05) [23].

Binary logistic regression analysis was used to examine the relationship between the dependent variable probability—assuming the value 1—and the independent variables. This method tested whether a relationship exists between the independent variables and a binary dependent variable. The dependent variable was defined as the general DiHA prescription (no=0, yes=1). Independent variables were selected based on the literature and were only included in the regression analysis if they met the following criteria [23]:

- For each group formed by categorical predictors, n≥25.
- Independent variables were not highly correlated with each other (r<0.7).

Since the regression model was based on well-founded theoretical considerations, we applied the “inclusion” method, meaning all variables within a block were entered into the model in a single step. All results are presented as odds ratios (ORs) with 95% CI.

### Ethical Considerations

The Ethics Advisory Board of Leipzig University reviewed the application for the research project using a simplified procedure and concluded that there were no ethical objections to its implementation.

## Results

The questionnaire was distributed to a total of 6035 HCPs, of whom 288 completely and 140 partially responded. After verifying and cleaning the data, 78 cases were excluded because no information was provided; the questionnaire was clicked through without being filled out. Ultimately, 350 HCPs participated in the survey, resulting in a response rate of 5.8%.

### Sociodemographics

Of the respondents, 45.9% (133/290) were male, and 53.1% (154/290) were female. Around a quarter of participants were aged 36-45 years (72/290, 24.8%), 46-55 years (86/290, 29.7%), and 56-65 years (82/290, 28.3%). The majority (278/290, 95.9%) were specialists, primarily in internal medicine (196/290, 63.6%). In terms of workplace settings, 42.5% (99/290) worked in general practice, and 35.6% (83/290) in specialist care. Additionally, 82.4% (238/289) held an additional qualification, most commonly in diabetology (63.8%). The largest group worked in hospitals (84/290, 29.3%), followed by group practices (80/290, 27.6%; Multimedia Appendix 2).

### Digital Affinity

HCPs rated their own digital affinity on a scale from 0 (not at all digitally affine) to 10 (very digitally affine), with an average rating of 6.80 (SD 2.03). Respondents who had already prescribed a DiHA or an HM-DiHA rated their own digital affinity with an average of 6.92 (SD 1.80) and 6.96 (SD 1.75), respectively, slightly higher compared to those who had not yet prescribed a DiHA or a DiHA from the indication area with 6.69 (SD 2.11) and 6.70 (SD 2.08). There was a correlation with a small effect size between general prescribing experience (η=0.057) and experience with prescribing HM-DiHA (η=0.051) and the respondents’ self-assessed digital affinity. HCPs’ own digital affinity did not correlate significantly with the intention to prescribe (ρ=0.156; *P*=.08).

Of the 292 respondents, 51.4% (150/292) had never used a health app as a patient, and this proportion was even higher for DiHA, with 86.6% (253/292) having never used one. A total of 34.6% (101/292) of HCPs had accessed manufacturer-provided test versions to evaluate a DiHA before prescribing it to patients. Among HCPs who prescribed a DiHA in general, more than half (75/137, 54.7%) had used manufacturer access, while 45.3% (62/137) had not; the correlation between general DiHA prescription and manufacturer access use was statistically significant (*V*=0.398; *P*<.001; Multimedia Appendix 3), with regression analysis further revealing that HCPs who had used manufacturer access were almost 12 times more likely to have prescribed a DiHA compared to those who had not (Table 1).

A statistically significant correlation with medium effect size was also found between an HM-DiHA prescription and manufacturer access use (*V*=0.330; *P*<.001), with 61.5% (72/117) of HCPs who prescribed a DiHA from this indication area having also used manufacturer access (Multimedia Appendix 3). There was a statistically significant correlation with medium effect size between the use of a DiHA as a patient (*V*=0.193; *P*=.05) and the use of manufacturer access (*V*=0.337; *P*<.001) with the intention to prescribe, with 30.9% (17/55) of HCPs who were more likely and 61.2% (41/67) who were likely to prescribe an HM-DiHA in the next 12 months having already used manufacturer access (Multimedia Appendix 4).

**Table 1:** Multivariate representation of the dependent variable “general digital health applications (DiHA) prescription,” categorized by independent variables. Details on assumption checks for the binary logistic regression are provided in Multimedia Appendix 6.

Independent variables		Regression coefficient (B)	*P* value	Odds ratio (95% CI)
**Gender (reference=male)**				
	Female	1.053	.01	2.867 (1.235-6.655)
**Activity within the framework of statutory health insurance care (reference=general practitioner care)**				
	Specialist care	–1.454	.002	0.234 (0.093-0.587)
**Additional title (reference=no)**				
	Yes	0.322	.58	1.38 (0.441-4.321)
**Patients treated per quarter (reference=<500)**				
	500-750	1.061	.16	2.89 (0.662-12.616)
	751-1000	1.548	.03	4.704 (1.148-19.274)
	1001-1500	2.447	.002	11.554 (2.472-54.012)
	1501-2000	1.153	.17	3.169 (0.616-16.301)
	>2000	2.138	.008	8.48 (1.738-41.384)
**Ever used a health app as a patient (reference=no)**				
	Yes	0.224	.63	1.251 (0.504-3.106)
**Ever used a DiHA as a patient (reference=no)**				
	Yes	–0.206	.76	0.814 (0.219-3.030)
**Ever used a DiHA manufacturer access (reference=no)**				
	Yes	2.459	<.001	11.689 (4.370-31.266)
Digital affinity (0=not at all digitally affine to 10=very digitally affine)		0.001	.99	1.001 (0.818-1.226)
Omnibus test of the model coefficients		N/A^a^	<.001	N/A
Cox and Snell *R*²		N/A	.37	N/A
Nagelkerke *R*²		N/A	.49	N/A

^a^N/A: not applicable.

### Experience With Previous DiHA Prescription

#### General DiHA Prescription

At 53.4% (187/350), more than half of the respondents had not yet prescribed a DiHA to their patients, with almost half of the respondents (89/187, 47.6%) named a lack of experience as the reason (multiple selection was possible). About one-fifth cited barriers such as insufficient efficacy evidence, low patient digital literacy, lack of interest in prescribing DiHA among HCPs, integration issues, time constraints, additional time requirements, and lack of training opportunities (Multimedia Appendix 5).

As part of an open-ended question, HCPs had the opportunity to provide additional reasons for not prescribing DiHA. The most common reasons included treating children and adolescents (15/64) for whom DiHA are not yet approved, or working in an inpatient setting (13/64), where prescribing a DiHA is either not possible or deliberately avoided due to the inability to ensure follow-up care. A lack of digital affinity, as well as insufficient information and knowledge (8/64), were also named.

Regarding the general DiHA prescription, statistically significant correlations with small to medium effect sizes were observed for the activity within the framework of SHI (*V*=0.481; *P*<.001), the use of an additional professional title (*V*=0.142; *P*=.05), the size of the city or municipality (*V*=0.231; *P*=.009), the working model (*V*=0.437; *P*<.001), and the number of patients treated per quarter (*V*=0.477; *P*<.001; Multimedia Appendix 3). Women were nearly 3 times more likely than men to have already prescribed a DiHA (OR 2.87, 95% CI 1.235-6.655; *P*=.01). HCPs treating 1001 to 1500 patients per quarter had 11.6 times higher odds (OR 11.55, 95% CI 2.472-54.012; *P*=.002) of having prescribed a DiHA, while those treating more than 2000 patients per quarter had 8.5 times higher odds (OR 8.48, 95% CI 1.738-41.384; *P*=.008), compared to HCPs treating fewer than 500 patients per quarter (Table 1).

#### Indication-Specific DiHA Prescription

**Figure 1:**
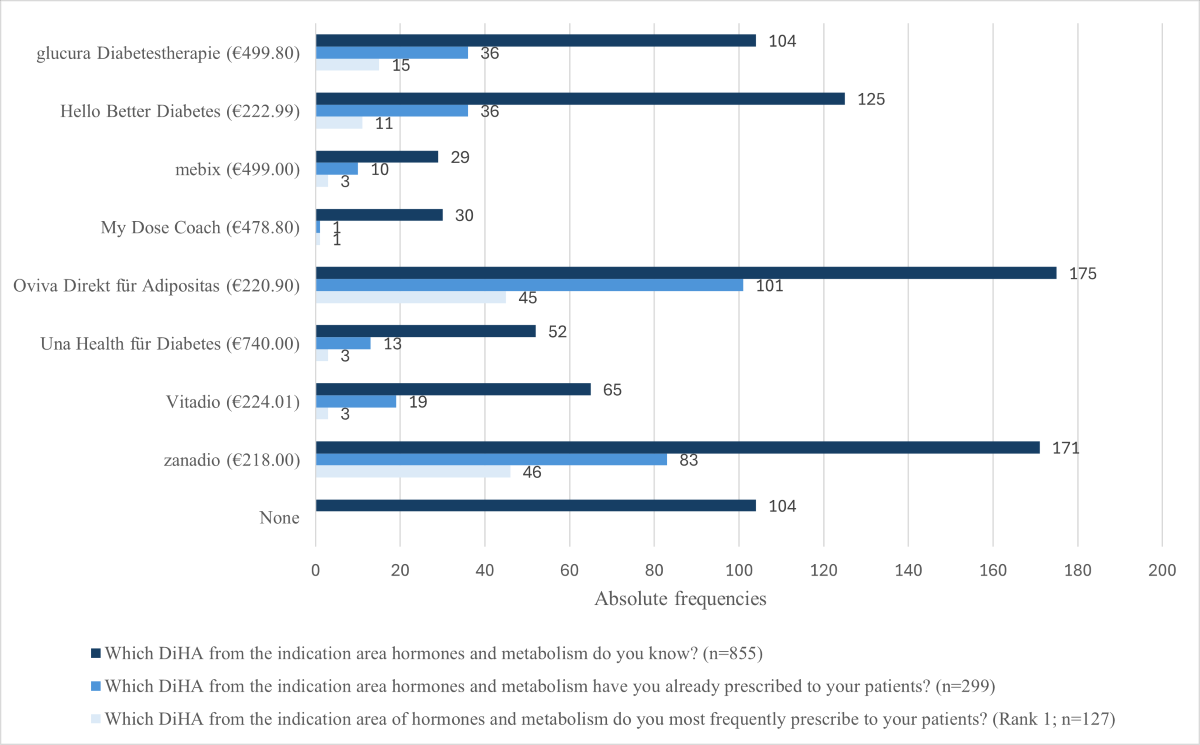
Experience with specific digital health applications (DiHA).

Of the HCPs who already prescribed a DiHA, the majority (139/163, 85.3%) had prescribed an HM-DiHA, while 14.7% (24/163) had not. Most HCPs were familiar with and frequently prescribed DiHA Oviva Direkt für Adipositas (n=175 familiar; n=101 prescribed) and zanadio (n=171 familiar; n=83 prescribed), while mebix (n=29 familiar; n=10 prescribed; the DiGA was removed from the DiGA directory on July 14, 2025, as no positive health care effect could be demonstrated) and My Dose Coach (n=30 familiar; n=1 prescribed; the DiGA My Dose Coach was removed from the DiGA directory on January 10, 2026, at the manufacturer’s request) were less known and infrequently prescribed. Overall, 104 HCPs were unaware of any DiHA in this area (Figure 1).

DiHA were most commonly prescribed for patients aged 36-45 years (n=54) and considered appropriate for all age groups, particularly 36-45 years (n=284) and 26-35 years (n=273), while least prescribed for those older than 65 years (n=4) and younger than 25 years (n=8).

### Prescription Frequency

**Figure 2:**
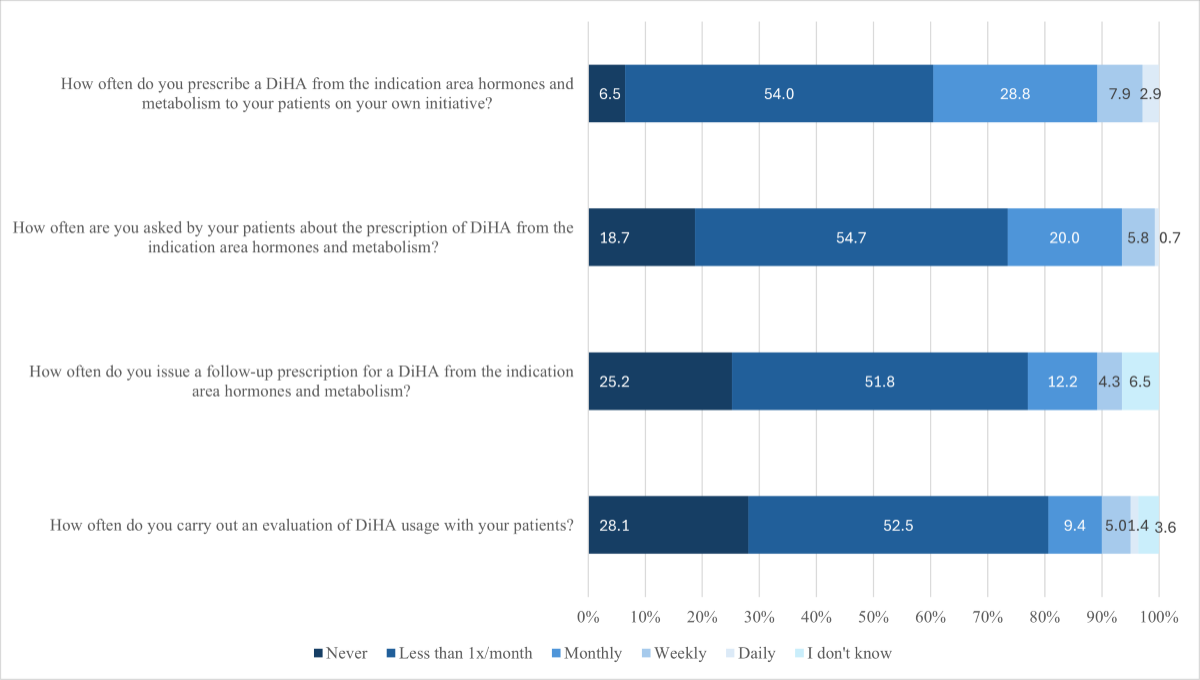
Experience with prescription frequency of digital health applications (DiHA).

More than half of the respondents had never (9/139, 6.5%) or less than once a month (75/139, 54%) prescribed an HM-DiHA on their own initiative. Similarly, three-quarters of HCPs had never been asked by patients to prescribe an HM-DiHA (26/139, 18.7%) or were asked less than once a month (76/139, 54.7%) for such a prescription. Follow-up prescriptions were also issued infrequently, with 35/139 (25.2%) never prescribing a follow-up and 72/139 (51.8%) prescribing them less than once a month (Figure 2). No statistically significant correlation was found between the frequency of prescribing DiHA on the HCPs’ own initiative and all the sociodemographic variables (Multimedia Appendix 7).

### Prescription Intention

Overall, 42.9% (149/348) of respondents were either very unlikely (83/348, 23.9%) or somewhat unlikely (66/348, 19%) to prescribe HM-DiHA in the next 12 months, while 40.5% (141/348) were either very likely (80/348, 23%) or somewhat likely (61/348, 17.5%) to do so. Additionally, 12.6% (44/348) of HCPs were undecided.

We found a statistically significant correlation between the intention to prescribe a DiHA in the next 12 months and activity within the framework of SHI (*V*=0.266; *P*<.001), with the majority (38/56, 67.9%) of those who were very likely to prescribe a DiHA being GPs, while only 23.2% (13/56) of specialists expressed the same likelihood. A statistically significant correlation was also identified between the intention to prescribe and the working model (*V*=0.246; *P*<.001), with 37% (20/54) of those rather unlikely and 40.3% (27/67) of those very unlikely to prescribe a DiHA working in hospitals (Multimedia Appendix 4).

### Health Care Effects

**Figure 3:**
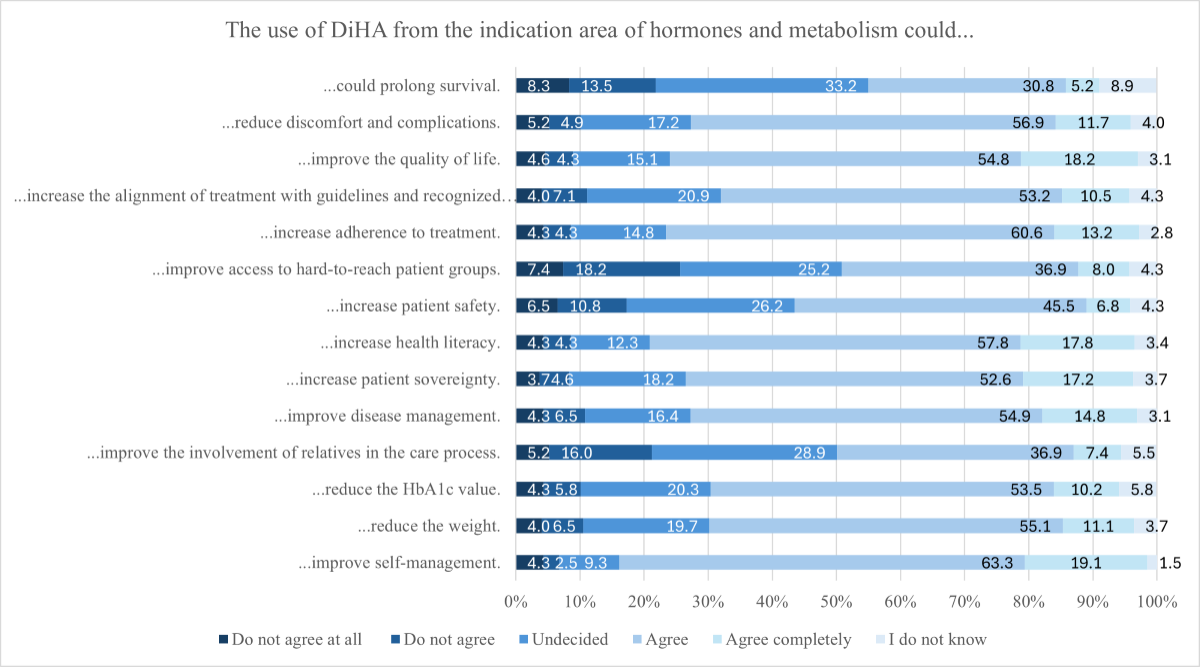
Potentially achievable positive health care effects of digital health applications (DiHA).

A total of 325 HCPs evaluated the potential positive health care effects that HM-DiHA could offer. The majority of respondents (267/325, 82.4%) indicated that these applications could enhance self-management (agree: 205/325, 63.3%; strongly agree: 62/325, 19.1%). Three-quarters of respondents believed that DiHA could improve health literacy, adherence, and QoL. The areas where the least potential for improvement was noted were in prolonging survival (agree: 100/325, 30.8%; strongly agree: 17/325, 5.2%), increasing the involvement of relatives in the treatment process (agree: 120/325, 36.9%; strongly agree: 24/325, 7.4%), and improving access to hard-to-reach patient groups (agree: 120/325, 36.9%; strongly agree: 26/325, 8%; Figure 3; Multimedia Appendix 8).

Apart from prolonging survival, improving access to hard-to-reach patient groups, and enhancing self-management, we found weak statistically significant correlations between individual potential positive health care effect assessment and experience with general DiHA prescriptions. Additionally, statistically significant correlations were observed between the assessment of potential positive health care effects—except for the involvement of relatives in the care process and improvement of self-management—and the frequency of prescriptions, as well as between the assessment of potential positive health care effects and the intention to prescribe (Multimedia Appendix 9).

**Figure 4:**
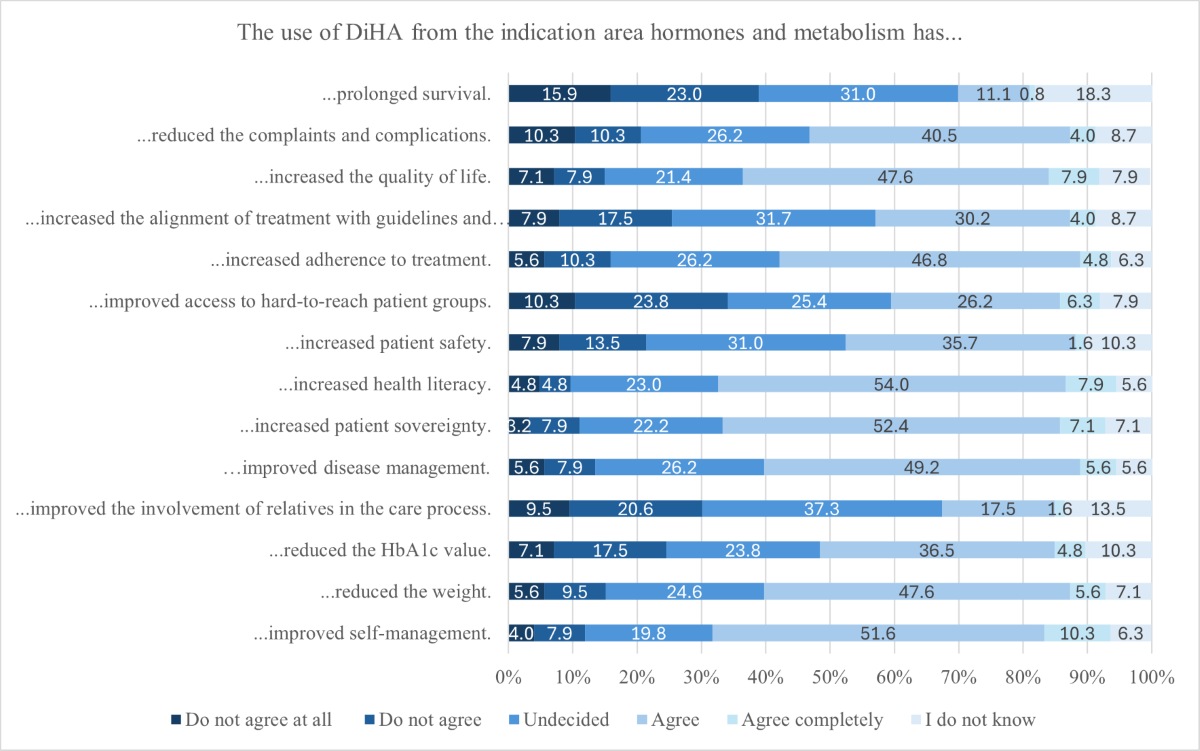
Actually observed positive health care effects of digital health applications (DiHA).

A total of 126 HCPs evaluated the positive health care effects that HM-DiHA have already achieved for their patients. Most respondents (78/126, 61.9%) reported that these applications had improved their patients’ self-management (agree: 65/126, 51.6%; strongly agree: 13/126, 10.3%) or health literacy (agree: 68/126, 54%; strongly agree: 10/126, 7.9%). More than half of the HCPs also observed improvements in their patients’ QoL, adherence, patient autonomy, disease management, and weight. The least positive health care effects were noted in relation to prolonging survival (agree: 14/126, 11.1%; strongly agree: 1/126, 0.8%) and increasing the involvement of relatives in the care process (agree: 22/126, 17.5%; strongly agree: 2/126, 1.6%; Figure 4; Multimedia Appendix 8).

We found weak statistically significant correlations between the assessment of the individual positive health care effects already achieved and the intention to prescribe, except for improvements in access to hard-to-reach patient groups. A medium, statistically significant correlation was observed between the intention to prescribe and the improvement in QoL as a perceived positive health care effect (ρ=0.723; *P*=.002; Multimedia Appendix 9).

### Barriers

**Figure 5:**
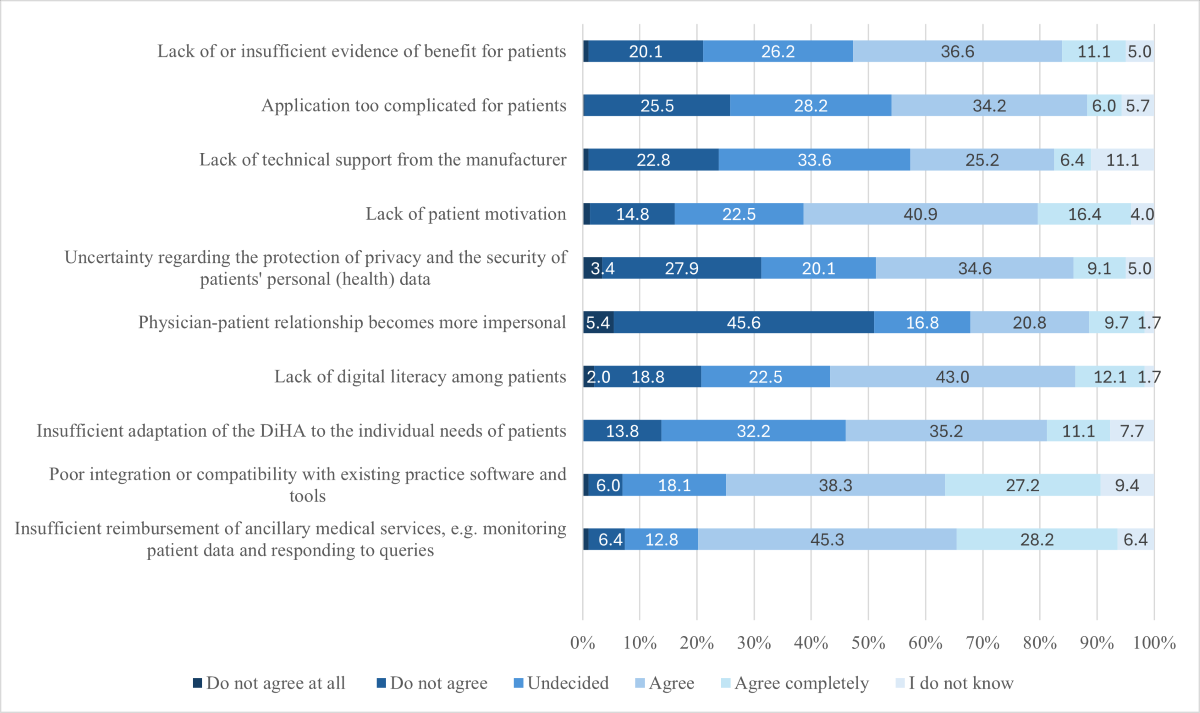
Barriers to digital health applications (DiHA) prescription.

A total of 298 HCPs evaluated the potential problems and barriers to prescribing HM-DiHA. Three-quarters of respondents identified the greatest barrier as the insufficient reimbursement for ancillary medical services, such as monitoring patient data or responding to queries (agree: 135/298, 45.3%; strongly agree: 84/298, 28.2%). More than half of the HCPs also cited barriers related to poor integration or compatibility with existing practice software, as well as a lack of digital affinity and motivation among patients. The lowest barriers were associated with an increasingly impersonal physician-patient relationship (agree: 62/298, 20.8%; strongly agree: 29/298, 9.7%) and a lack of technical support from the manufacturer (agree: 75/298, 25.2%; strongly agree: 19/298, 6.4%; Figure 5; Multimedia Appendix 10).

Weak, statistically significant correlations were found between the assessment of barriers—such as a lack of or insufficient proof of benefit for patients (*V*=0.247; *P*=.003), a lack of technical support from the manufacturer (*V*=0.195; *P*=.046), poor integration or compatibility with existing practice software (*V*=0.241; *P*=.004), and insufficient reimbursement for ancillary medical services (*V*=0.223; *P*=.001)—and the general DiHA prescription. No statistically significant correlations were observed regarding potential barriers and the prescription of HM-DiHA, nor with prescription frequency, except for the insufficient adaptation of DiHA to the individual needs of patients (ρ=–0.382; *P*<.001). However, numerous statistically significant, negative correlations were identified between the perceived barriers and the willingness to prescribe within the next 12 months (Multimedia Appendix 11).

There was also an option to answer an open-ended question to provide additional problems and barriers, which 43 HCPs used. Financial aspects were the most frequently mentioned here. On one hand, HCPs considered their own remuneration for prescribing DiHA insufficient; on the other hand, they also found the costs of DiHA too high. Evidence was also cited as a barrier, with respondents pointing to the lack of long-term studies and the absence of patient feedback regarding the effectiveness of DiHA. The acceptance and motivation of patients were another challenge. Despite initial motivation, many patients discontinue DiHA use. Additionally, there is often a lack of basic acceptance of digital interventions or sufficient digital competence (particularly among older patients), which is a fundamental prerequisite for their use. Other challenges mentioned were related to the HCPs' digital affinity and their knowledge of DiHA. This includes a lack of awareness about the existence of these applications, insufficient information about DiHA services and how they work, their suitability for specific patient groups, and a lack of training opportunities.

### DiHA Evidence

**Figure 6:**
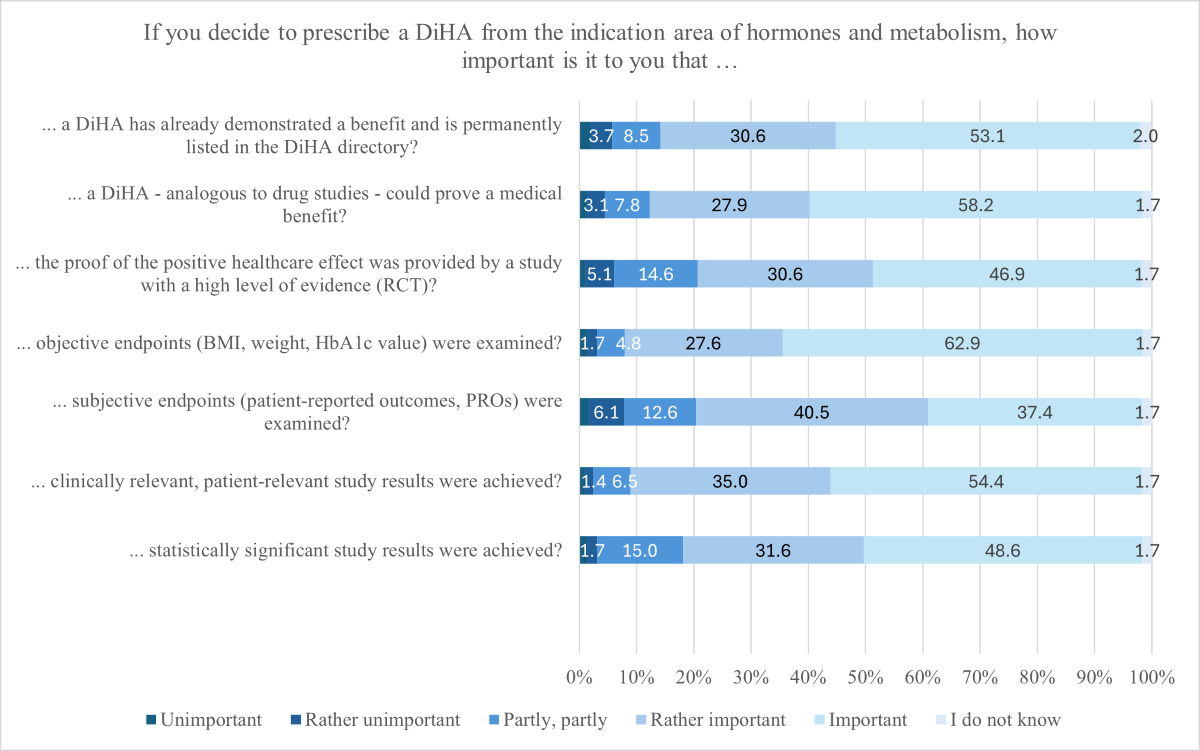
Digital health applications (DiHA) evidence.

A total of 294 HCPs answered the questions to assess the relevance of the DiHA evidence. With regard to the evidence of DiHA, the HCPs consider it most important that objective end points such as BMI, weight, or HbA_1c_ value were examined (rather important: 81/294, 27.6%; important: 185/294, 62.9%), that clinically relevant, patient-relevant study results were achieved (rather important: 103/294, 35.0%; important: 160/294, 54.4%), that the DiHA—analogous to drug trials—was able to demonstrate a medical benefit (rather important: 82/294, 27.9%; important: 171/294, 58.2%) and that the DiHA has already demonstrated a benefit and is permanently listed in the DiHA directory (rather important: 90/294, 30.6%; important: 156/294, 53.1%; Figure 6).

## Discussion

### Overview

To date, no research in scientific literature explores the prescribing experiences, as well as the potential and challenges of HM-DiHA, from the perspective of HCPs. Therefore, we conducted a quantitative data collection (n=350) in collaboration with the GDA to address this research gap. The primary aim of the study was to explore the experiences HCPs have had with prescribing DiHA in general, and specifically in the area of hormones and metabolism, as well as to assess their prescription intention, and to identify the positive health care effects and barriers associated with DiHA use and prescription. Based on the findings, recommendations for action were to be developed to enhance the acceptance of DiHA among HCPs.

### Experience With Previous DiHA Prescription

Our results show that DiHA have not fully arrived in statutory standard care yet—although they can be prescribed since 2020—as more than half of the respondents (187/350, 53.4%) have not yet prescribed DiHA in general. It should be noted that the survey participants likely represent a sample generally more open to digitalization, which could indicate an even lower adoption rate in the broader population. However, compared to previous surveys, this figure has risen significantly. In these earlier surveys, the proportion of participants who had already prescribed a DiHA was 10% (103/1299; period: 2020-2021) [15], 14% (536/3829; time point: 2022) [16] and 59.4% (58/97; time point: 2023) [18]. However, the results of Brecher et al [18] also show that half of those GPs who had already prescribed a DiHA only did so less than once a month (39/97, 49%). This proportion is comparable to that in this study, which was 54% (75/139). Overall, the scientific literature shows that there is still a high degree of skepticism on the part of HCPs regarding DiHA [16,25].

Wangler and Jansky [16] showed that HCPs in urban settings (21%) were statistically significantly more likely to prescribe DiHA compared to HCPs who worked in rural areas (5%). This study also showed that there was a statistically significant correlation between the general prescription of a DiHA and the size of the municipality or city. Of the HCPs who had already prescribed a DiHA, 26.5% (36/136) worked in a municipality or city with 5000-20,000 or more than 500,000 inhabitants. Overall, DiHA prescription is considered suitable for all age groups, with people aged 26-35 years and 36-45 years being cited most frequently. Dahlhausen et al [15] also found that 40.7% (527/1295) of respondents would primarily prescribe DiHA to younger patients. Some HCPs have identified a lack of DiHA options for children and adolescents, suggesting that future development should focus on their specific needs and requirements. Studies highlight the potential of digital health interventions in treating children and adolescents with obesity and DM and advocate for their integration into existing treatment approaches [26,27]. Among HCPs who prescribed a DiHA in general, those who used manufacturer access were nearly 12 times more likely to have prescribed a DiHA, highlighting that targeted provision of such access can enhance DiHA acceptance.

### Prescription Intention

Although our results showed a positive correlation between the numerous positive health care effects already perceived and the intention to prescribe, 42.9% (149/348) of respondents did not intend to prescribe DiHA in the next 12 months. This represents an increase compared to previous surveys, in which 37% (19/51; time point: 2020) [14], 30.3% (393/1299; time period: 2020-2021) [15], 13% (498/3829; time point: 2022) [16], and 38.5% (37/97; time point: 2023) [18] reported similar intentions. Differences in study periods, survey methodologies, and target populations may have contributed to this variance. The proportion of respondents who remain uncertain about prescribing DiHA (44/348, 12.6%) has slightly decreased compared to Dahlhausen et al (259/1299, 19.9%) [15].

These results suggest that further research is needed to identify key factors influencing DiHA acceptance and, in turn, to develop measures that enhance acceptance among HCPs in the field of hormones and metabolism. To sustainably implement DiHA in SHI standard care, the GDA has already outlined initial approaches aimed at making HM-DiHA an integral part of existing and future disease management programs [28]. Obesity-related DiHA are already included in the medical guideline on “Prevention and Treatment of Obesity” [29]. However, integrating diabetes-related DiHA into current guidelines has not occurred yet. DiHA with proven benefits specifically for diabetes should be more firmly embedded in the treatment process through integration into medical guidelines, allowing both HCPs and patients to recognize their direct added value. This recommendation is further supported by Wangler and Jansky’s [30] findings. They showed that diabetes specialists would generally be more willing to include health apps in patient care (26% much more willing; 54% somewhat more willing) if national care guidelines for diabetes specifically addressed the use of such interventions.

Apart from the activities within the framework of SHI and the working model, the sociodemographic characteristics of HCPs did not correlate with the intention to prescribe. There is also disagreement within the scientific literature regarding the influence of sociodemographic characteristics, such as age and gender, on the acceptance of digital interventions. Some studies have identified these factors as determinants [31], while others have not [32]. However, the working model impacts the intention to prescribe, with the majority of HCPs working in hospitals being either rather unlikely (20/54, 37%) or very unlikely (27/67, 40.3%) to prescribe DiHA. Until March 2022, DiHA were only used in outpatient care and could not be prescribed in the inpatient sector due to the lack of adjustments to the discharge management framework agreement. However, our results show that some HCPs still believe they cannot prescribe DiHA or choose not to do so due to the lack of follow-up care because they work in the clinical setting. In the inpatient setting, more information should be provided about the possibilities for prescribing DiHA and monitoring their progress.

### Health Care Effects

Our results indicate that the majority of HCPs recognize both the potential positive health care effects that DiHA use can achieve and the positive health care effects that have already been realized through its use. A positive attitude toward DiHA and their acceptance among HCPs can only be achieved if HCPs perceive the potential benefits and advantages of DiHA use for their patients, further supported by findings that an HCP’s willingness to prescribe is influenced by their own perception of the usefulness of a digital intervention [33]. Therefore, the positive health care effects and potential of DiHA should be transparently presented and made comparable through standardized quality indicators.

Most respondents have observed improvements in patient-reported outcomes (PROs) such as self-management, health literacy, QoL, adherence, patient autonomy, disease management, and weight management as a result of DiHA use. These findings emphasize the high relevance of PROs and align with those of Dahlhausen et al [15], who reported improvements in adherence (997/1294, 77%), health literacy (842/1294, 65%), and disease management (783/1294, 60.5%). Similarly, Wangler and Jansky [16] found improvements in compliance (95%), mobility (94%), health awareness and education (93%), and self-management (91%) as the most common health care effects. Other studies also support this perspective, confirming the improvement of these PROs through DiHA use [25].

The direct patient benefits of HM-DiHA have already been confirmed for those permanently listed in randomized controlled trials [12,13,34]. Initial trends regarding patient benefits have also been observed for DiHA that are in the preliminary listing stage [10,35]. For example, diabetes-related DiHA have demonstrated reduced HbA_1c_ levels, while obesity-related DiHA have shown decreased weight as part of their primary end points. However, our results reveal that only 41.3% (52/126) of HCPs observed a reduction in HbA_1c_ levels in their patients, and 23.8% (30/126) were unsure about this outcome. Regarding weight reduction, 53.2% (67/126) of HCPs observed a decrease, while 24.6% (31/126) were unsure. To strengthen HCPs’ acceptance and confidence in the effectiveness of DiHA, manufacturers should demonstrate the sustained positive health care effects observed in studies with a high level of evidence. Additionally, they should consider including factors such as patient adherence and the intensity of use in their analyses.

### Barriers

#### Overview

The high proportion of HCPs who are very unlikely (83/348, 23.9%) or somewhat unlikely (66/348, 19%) to prescribe HM-DiHA over the next 12 months or remain uncertain (44/348, 12.6%), can be attributed to the identified barriers. Given the established correlation between perceived barriers and the willingness to prescribe, it is crucial to specifically address and overcome these obstacles.

#### Barrier 1 (Financial Barrier): The Reimbursement for Accompanying Medical Services Related to the DiHA Prescription is Inadequate

Insufficient reimbursement for ancillary services, such as monitoring patient data or responding to queries, was identified as the main barrier by nearly three-quarters of respondents (219/298, 73.5%), compared with half of HCPs in a recent study [15]. Managing DiHA is seen as an additional responsibility without additional remuneration, so HPCs lack financial incentives to engage with DiHA.

Monetary factors, such as reimbursement and the costs of digital interventions, are among the strongest predictors of HCPs’ acceptance of digital interventions [32,33]. In particular, the long-term costs of the technology, as well as the costs of devices and applications [33]. Financial incentives for prescribing medical services [36], including those related to DiHA [15], can enhance HCPs’ acceptance.

In Germany, the uniform value scale (Einheitlicher Bewertungsmaßstab), which includes a fee schedule, regulates DiHA remuneration. For permanently listed obesity DiHA, an additional flat rate of 64 points (€7.64; US $8.9) is available for monitoring and evaluation. However, this can only be billed once per treatment or illness. There are no fee schedule items for the permanently listed diabetes DiHA. The reimbursement for follow-up checks of provisionally listed DiHA is significantly lower than the standard flat rate for personal or video consultations in primary care [37]. This highlights a clear imbalance and may act as a barrier to DiHA adoption among HCPs.

Overall, our findings highlight the significant importance of reimbursing medical services related to DiHA prescriptions, with HCPs rating the current reimbursement as insufficient. To support the sustainable use of DiHA, clearly defined and adequately incentivized billing codes are needed—ones that realistically reflect the time and effort required by HCPs. Initial steps, such as the introduction of the uniform flat rate, mark a move in the right direction but remain insufficient for widespread implementation. A targeted refinement of the remuneration framework is therefore essential to embed DiHA not only technically, but also economically, into routine medical practice.

#### Barrier 2 (Financial Barrier): The Costs for a DiHA Are Too High

Another barrier raised by numerous HCPs was the perceived high cost of individual DiHA. In the first year after a DiHA is included in the DiHA directory, the manufacturer sets the actual price, regardless of whether the DiHA is listed preliminarily or permanently. To prevent arbitrary pricing by manufacturers, this price is capped by a maximum amount regulation, under which the National Association of SHI Funds (GKV-Spitzenverband) and the manufacturers’ associations reach a joint framework agreement and set maximum amounts for 90-day DiHA usage. At the end of the first 12 months of a DiHA’s listing, the reimbursement amount is negotiated between the GKV-Spitzenverband and the manufacturers. From January 1, 2026, at least 20% of the permanent remuneration amount for a DiHA will be performance-related, depending on its successful application. Success is determined through the “application-related performance measurement,” the specific form of which is currently under discussion. To increase the acceptance of DiHA and the willingness to prescribe them, HCPs should be more involved in the entire pricing process for DiHA, as well as in the development of the application-related performance measurement.

Currently, the prices of HM-DiHA are generally higher compared to other indication areas [38], with prices ranging from €218 (US $255; zanadio) to €740 (US $867; Una Health for diabetes) [39]. A comparison over recent years reveals that the most significant increase—20%—occurred between 2021 and 2022. Since then, manufacturer prices have risen by an additional 11%. Overall, prices increased by 39%, equivalent to €162 (US $190), between 2020 and 2024 [40]. The cost of treatment significantly influences the prescribing decisions of HCPs [36], which is supported by the results of this study, with cheaper DiHA being prescribed more often compared to more expensive DiHA. However, prices should be structured in a way that does not overburden the health care system, which is financed on a solidarity basis, while still offering sufficient incentives for DiHA manufacturers to invest in the design and improvement of their products.

Powell and Torous [41] outlined a patient-centered framework for evaluating the economic value of the clinical benefits of DiHA. They proposed a model incorporating factors such as the country-specific value of quality-adjusted life years, engagement rates, and the app’s health impact. By applying this model to 2 DiHA, the authors aim to provide country-specific estimates of their economic value, helping to guide future research in digital health. This approach can inform value-based payment models and clinical decision-making [41].

While health economic evaluations typically rely on incremental cost-effectiveness ratios—which represent the difference in cost between the intervention group and the control group divided by the difference in their effects—applying this approach in the DiHA context may be challenging, as German health authorities are fundamentally opposed to the use of general cost-effectiveness thresholds. Furthermore, deriving an appropriate incremental cost-effectiveness ratio through an alternative approach would necessitate the consideration of all relevant treatment options within the therapeutic area, making it a highly time-consuming process [42]. Gensorowsky et al [42] argued that a pragmatic approach to DiHA pricing could involve aligning their demonstrated benefits with the cost-effectiveness benchmarks of established SHI-covered treatments in the same therapeutic area. Therefore, a DiHA price would be considered appropriate if the cost per unit of benefit generated by the DiHA is equal to or lower than the cost per unit of benefit provided by the existing therapy [42]. Going forward, the evaluation of a DiHA should not only encompass its positive health outcomes but also include a comprehensive health economic analysis.

#### Barrier 3 (Acceptance and Motivation): There Is a Lack of Acceptance, Digital Affinity, and Patient Motivation

Additional barriers include patients’ lack of motivation (171/298; 57.3%) and limited digital affinity (164/298; 55.1%). Many patients either do not accept DiHA or have insufficient digital literacy, particularly older individuals, as reflected by most respondents reporting that patients never requested a DiHA (26/139; 18.7%) or requested one less than once a month (76/139; 54.7%). Similarly, findings from Wangler and Jansky indicate that GPs are either never (41%) or only occasionally (18%) approached by patients regarding DiHA [16]. A survey of diabetes specialists further supports this, revealing that patients never (10%) or only rarely (38%) inquire about health apps for the prevention and management of T2DM [17].

Moreover, our survey revealed that patients frequently discontinue DiHA use, which may explain why follow-up prescriptions are never issued (35/139; 25.2%) or issued less than once a month (72/139; 51.8%). A systematic review on the acceptance of mHealth interventions among HCPs also identified patients’ digital affinity and motivation as key factors influencing acceptance [33]. These findings highlight the critical importance of educational efforts by HCPs to enhance patient engagement and sustained use of DiHA.

#### Barrier 4 (Evidence-Based Research and Data): There Is a Lack of Study Evidence and Long-Term Data

Nearly half of the HCPs surveyed (142/298, 47.7%) cited a lack or insufficiency of evidence for patient benefits as a barrier, consistent with Dahlhausen et al [15] (712/1298, 54.9%). At the same time, evidence related to DiHA—such as the inclusion of objective end points, the demonstration of clinically and patient-relevant outcomes, proof of medical benefit, and the resulting permanent listing in the DiHA registry—plays an important role for HCPs when deciding whether to prescribe an HM-DiHA. One contributing factor is the lack of patient feedback, which makes it difficult to assess the overall health care impact of DiHA. Additionally, the absence of long-term studies prevents a thorough evaluation of its sustained benefits. Given this context, it is crucial to ensure that all available evidence on the mechanisms of action and proven benefits of DiHA is easily accessible, standardized, and transparent—starting with the DiHA directory at the BfArM.

Even within the DiHA directory, information relevant to prescription decisions is often presented incompletely [43], despite being considered an essential tool for information and decision-making [14]. Wangler and Jansky [16] also highlight that the DiHA directory is viewed critically and lacks sufficient detail. Currently, the decision on whether a DiHA is granted preliminary or permanent inclusion in the directory is made solely by the BfArM. However, for future DiHA classified as risk class IIb—where the potential hazards are higher compared to lower-risk applications—HCPs should be involved in the approval process at an early stage. Furthermore, manufacturers should continue evaluating the efficacy of their DiHA even after permanent listing in the directory to address the need for more long-term data.

In accordance with the application-related performance measurement, patient adherence and the usage intensity of each application should also be considered [44]. It is important to acknowledge that adherence data, while offering an indication of users’ engagement with the DiHA, do not necessarily capture the positive health care effect achieved. Although higher usage frequency may be associated with improvements in health status, it does not guarantee that the intended clinical end points are met. Consequently, for the design of application-related performance measurement, adherence should be interpreted only as a limited proxy for effectiveness. A more robust assessment requires combining adherence metrics with direct clinical end points, PROs, or other objective health indicators.

In addition, increasing the involvement of HCPs in the development of DiHA could help mitigate feelings of exclusion and disconnection from what is often perceived as a nonmedically driven development process [45]. Moreover, participatory technology development—aligned with a user-centered design approach—has the potential to enhance the long-term use and effectiveness of DiHA [46].

#### Barrier 5 (Knowledge and Information): There Is a Lack of Information and Knowledge Among HCPs

The lack of information and knowledge regarding the basic existence of DiHA, as well as their specific content, functionality, and suitability for certain patient groups, presents a significant challenge. In a recent study, the lack of information was identified as the greatest barrier (1135/1299, 87.4%) probably because the DiHA system was only recently introduced in Germany at the time of the study [15]. Even 3 years after the DiHA system was implemented, only 38.1% of GPs felt sufficiently informed to prescribe DiHA [18]. From an information management perspective, this persistent information gap illustrates deficits in the systematic provision, dissemination, and integration of knowledge relevant to DiHA. Effective information management in health care entails not only ensuring that accurate and relevant information is available, but also that it reaches the appropriate stakeholders at the right time and in an actionable format. The current situation reflects a lack of coordinated information governance and highlights the absence of an infrastructure that supports timely, user-oriented knowledge distribution for HCPs. To address future information deficits, there should be widespread education on the function and benefits of DiHA, as well as their prescription options. This educational effort should be as comprehensive as possible, involving the BfArM, the GKV-Spitzenverband, SHI physicians’ associations, the state and federal medical associations, the manufacturers of DiHA, medical societies, and obesity and diabetes networks. In this context, these actors collectively form the core of a decentralized information management system whose collaboration is crucial to improving the flow, accessibility, and quality of information in the German DiHA system.

The role of medical associations should be emphasized here, as a survey of diabetes specialists revealed that 73% of respondents rely on the GDA for information on the evaluation and recommendation of mobile health apps [17]. The current body of research indicates that training can help fully leverage the potential of digital interventions. HCPs often fail to use the full range of features offered by digital interventions simply because they are unfamiliar with them [47]. Additionally, GPs would appreciate both written and in-person training on DiHA, along with support for its implementation in daily practice [14]. For this reason, training programs tailored to the target audience should be developed and made accessible. In this context, incentives for training—such as certification for continuing medical education—could be beneficial [15,16].

### Limitations

The authors developed the online questionnaire based on preliminary work and a literature review. It was essential to develop a dedicated questionnaire for the DiHA survey targeting HCPs in the field of hormones and metabolism, as this specialty faces specific challenges and requirements in the use of DiHA. A tailored questionnaire allowed for a focused exploration of the unique needs and experiences of these HCPs, particularly regarding the integration of DiHA into the treatment of complex hormonal and metabolic conditions.

While the existing scientific evidence was thoroughly identified, no systematic literature search was conducted. Additionally, the questionnaire underwent multistage pretesting, which helped to enhance its validity. An online survey offers an advantage through quick, contactless collection of large volume of data, ensuring anonymity and minimizing social desirability bias. However, a drawback is that any queries could not be addressed directly during the survey; instead, they could only be answered via email after the survey was completed.

The questionnaire link was distributed via email to all HCPs registered with the GDA. This one-sided recruitment method introduces a potential bias, which should be considered when evaluating the representativeness of the results. However, it allowed for nationwide distribution of the survey in Germany, thus increasing the relevance of the findings for the country. The distribution of respondents was balanced, with 46.6% (163/350) having already been prescribed a DiHA and 53.4% (187/350) not having done so yet. It cannot be ruled out that HCPs with a stronger interest in digital interventions were more likely to participate in the survey, and thus, potential response bias should be considered when interpreting the results.

The response rate of 5.8% (350/6,035) in this study is lower than in previous studies on physicians’ attitudes toward DiHA without a specific indication focus (6.4% [14]; 7% [15]; 28% [16]; 31% [17]; 48.8% [18]). A total of 78 cases had to be excluded due to missing data. Additionally, further missing values were identified during the subsequent questionnaire processing, resulting in a final sample of 290 HCPs who completed the survey in full. Given this response rate, the survey cannot claim to be fully representative. Before data analysis, the missing values were examined for patterns, but no correlations were found. Imputation procedures were deliberately avoided, as the missing values were not random but rather due to the deliberate nonresponse of the HCPs.

A major limitation in data analysis arose from the sample size, which reduced the statistical power of the tests and increased the risk of a type II error (beta error). This means that potentially existing effects within the population may not be detected [23]. To address this, we used binary logistic regression analysis to investigate potential causal relationships. This approach has relatively few requirements (eg, for each group formed by categorical predictors, n≥25; and independent variables must not be highly correlated, *r*>0.7) and is comparatively insensitive to outliers [23]. The omnibus test of the model coefficients confirmed the significance of the regression model and its overall performance. The Cox and Snell *R*² of 0.371 and Nagelkerke *R*² of 0.497 indicated adequate model quality. Additionally, the Hosmer-Lemeshow test (*χ*²_8_=3.713; *P*=.88) revealed only minor deviations between the predicted and observed values, which was also confirmed by the overall prediction accuracy (predicted vs observed) of 80.3%. Nevertheless, the CIs are very wide in some cases, which could be due to the sample size. If the number of observations is low, particularly in one category of the dependent variable, this can result in less reliable CIs and, consequently, more uncertain estimates [23].

### Conclusions

For more than 5 years, DiHA have been driving innovation in patient care, offering new solutions for both patients and HCPs while helping to bridge existing gaps in care. Physicians play a crucial role in integrating DiHA into standard care. The aim of our study was to gather insights from HCPs regarding their experiences with prescribing DiHA, their prescribing intentions, as well as the opportunities and challenges associated with HM-DiHA. Our results indicate that DiHA have not yet been fully integrated into standard care, highlighting the need for targeted measures to facilitate their adoption in the coming years. Based on our findings, we provide key recommendations for health policymakers, DiHA manufacturers, and DiHA prescribers. While these recommendations are strong and actionable, it is important to note that the findings are exploratory, cross-sectional, and based on a limited sample size, and should therefore be interpreted with appropriate caution.

1. Development of DiHA tailored to children and adolescents’ specific needs and requirements.
2. Targeted provision of DiHA manufacturer access.
3. Strengthening the integration of DiHA into medical guidelines would help anchor it more firmly within the treatment process.
4. Information on the options for prescribing DiHA and monitoring their progress in inpatient settings.
5. Transparent presentation of positive health care effects and development of standardized quality indicators for their comparability.
6. Adequate compensation for accompanying medical services.
7. Inclusion of HCPs in the entire pricing process for DiHA, and the design of application-based performance measurement.
8. Health economic evaluation of DiHA.
9. Early involvement of HCPs in the approval process, particularly for DiHA with risk class IIb.
10. Ongoing effectiveness evaluation, even after permanent inclusion in the directory.
11. Comprehensive information and certified training on the functions, benefits, and prescription options of DiHA.

Furthermore, indication-specific research is needed to validate the findings and adapt them to other medical conditions. Future studies could also benefit from exploring the potential and challenges of DiHA from the perspective of HCPs across different countries and health care systems.

## Appendix

Multimedia Appendix 1 Survey instrument.

Multimedia Appendix 2 Distribution of the main sociodemographic characteristics of the health care providers.

Multimedia Appendix 3 Experience with previous digital health application prescriptions in relation to sociodemographic variables, n (%).

Multimedia Appendix 4 Prescription intention in relation to sociodemographic variables, n (%).

Multimedia Appendix 5 Reasons for the lack of digital health application prescription.

Multimedia Appendix 6 Binary logistic regression analysis: general digital health application prescription and sociodemographic variables/digital affinity.

Multimedia Appendix 7 Prescription frequency in relation to sociodemographic variables, n (%).

Multimedia Appendix 8 Assessment of potential (N=325) and actual (n=126) healthcare effects, n, (%).

Multimedia Appendix 9 Correlation between healthcare effects and prescription experience, prescription frequency and prescription intention.

Multimedia Appendix 10 Assessment of barriers (n=298) for digital health application prescription, n (%).

Multimedia Appendix 11 Correlation between barriers and prescription experience, prescription frequency and prescription intention.

## Abbreviations

BfArM: Federal Institute for Drugs and Medical Devices [Bundesinstitut für Arzneimittel und Medizinprodukte]
DiHA: digital health applications
DM: diabetes mellitus
GDA: German Diabetes Association
GP: general practitioner
HbA1c: glycated hemoglobin
HCP: health care provider
HM-DiHA: hormones and metabolism digital health applications
ICD-10: International Statistical Classification of Diseases, Tenth Revision
OR: odds ratio
PRO: patient-reported outcome
QoL: quality of life
SHI: statutory health insurance
T2DM: type 2 diabetes mellitus

## Data Availability

This study is based on primary data collected through a quantitative online survey, conducted with the support of the German Diabetes Association (Deutsche Diabetes Gesellschaft). The dataset generated and analyzed during this study is not publicly available due to participant privacy but may be made available from the corresponding author upon reasonable request.
