# Supplementary material for: Prescribing Experiences, Potentials, and Challenges of Digital Health Applications in the Field of Hormones and Metabolism: Cross-Sectional Survey Study of Health Care Providers in Germany
Source: JMIR Form Res. 2025 Dec 31;9:e77792. doi: 10.2196/77792 (PMC12805319; doi:10.2196/77792)
Supplement: Multimedia Appendix 1 [file formative_v9i1e77792_app1.docx]

Multimedia Appendix 1: Survey Instrument

### Introduction

Dear participants,

as part of my doctorate at the University of Leipzig, I am conducting a survey of healthcare professionals on digital health applications (DiHA) from the indication area of hormones and metabolism. As low-risk medical devices, DiHA can be prescribed for people with statutory health insurance since September 2020, which is why the target group of healthcare professionals plays a decisive role in the implementation process. All healthcare professionals can take part in the survey - regardless of whether they have already prescribed a DiHA (from the indication area hormones and metabolism) or not.

The aim is to analyze (1) the experience and willingness to prescribe, (2) the potential and actual healthcare effects, (3) the barriers, (4) the assessment of the study evidence in each case regarding DiHA from the indication area of hormones and metabolism and (5) the digital affinity of the healthcare professionals.

Completing the survey takes about 5 to 7 minutes.

Thank you very much for your participation, for your support of my dissertation and for your contribution to the research!

Melanie Mäder

melanie.maeder@uni-leipzig.de

### Explanation DiHA


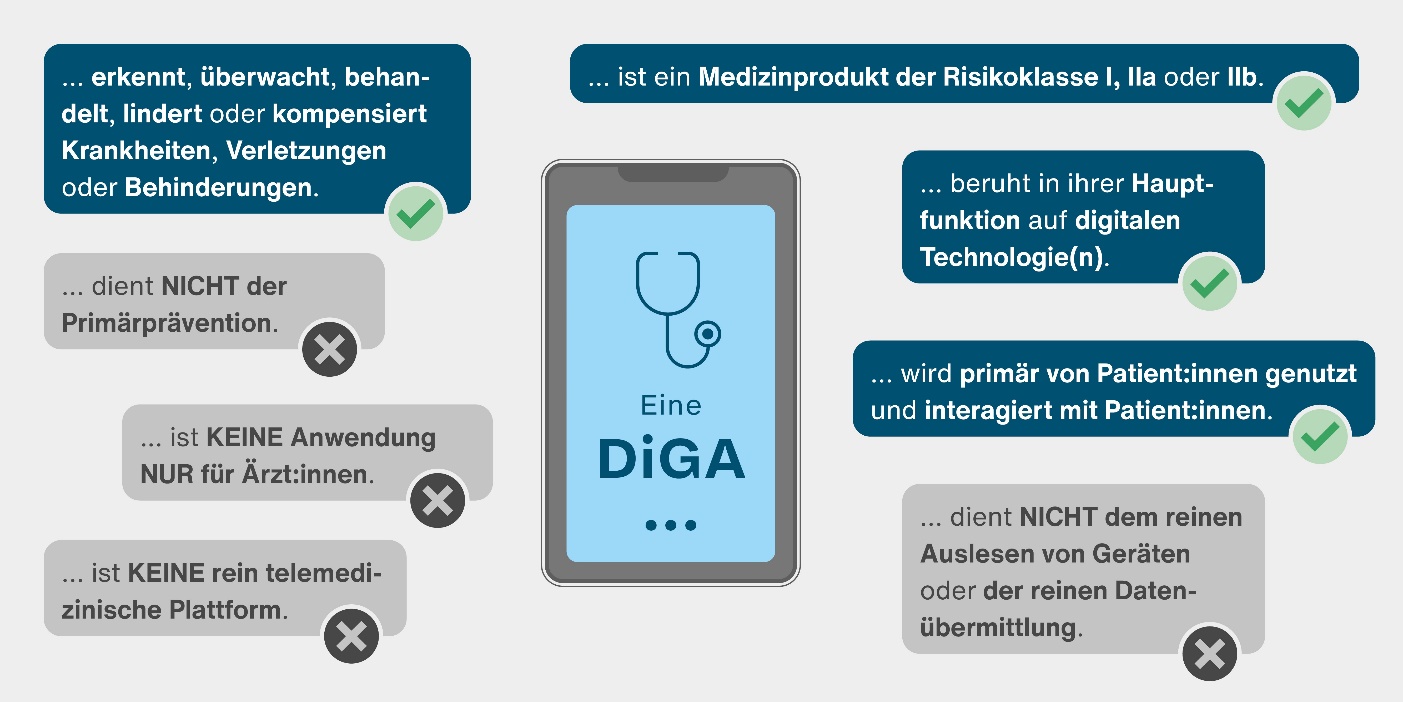


### Data protection

Information on data collection, storage and destruction:

- Your participation in the survey is voluntary. You can cancel the questionnaire at any time.
- Your participation is anonymous, so your answers cannot be traced back to you. This also means that your personal data record cannot be identified by us once the survey has been completed.
- The data collected will be used exclusively for scientific purposes.
- Unauthorized third parties have no access to the data itself or to its evaluation.
- The raw data will be destroyed after a storage period of five years after the end of the project.
- The research does not pursue any commercial interest. All data will be treated in strict confidence.
- Consent can be withdrawn at any time without giving reasons and without any associated disadvantages.

Checkbox: To start the survey, please accept the privacy policy.

### Experience and willingness to prescribe

The following is about your **experience** with DiHA in general, with DiHA from the indication area hormones and metabolism as well as your **willingness to prescribe** DiHA from the indication area hormones and metabolism.

| No. | Question | Answer | Coding |
| --- | --- | --- | --- |
| 1  E01 | Have you ever prescribed a **DiHA** to your patients? | Yes  No | 1  2 |
| 2  E02 | Have you ever prescribed a **DiHA from the indication area of hormones and metabolism** to your patients? | Yes  No | 1  2 |
| *#1 no - continue with #3, #7, #12, #13, #14*  *Combination #1 yes and #2 no - continue with #3, #7, #12, #13*  *Combination #1 yes and #2 yes - continue with #3 - #13* | | |  |
| 3  E03 | Which DiHA from the indication area hormones and metabolism **do you know**? [Multiple choice] | glucura Diabetestherapie  HelloBetter Diabetes und Depression  Mebix  My Dose Coach  Oviva Direkt für Adipositas  Una Health für Diabetes  Vitadio  Zanadio  None  Others | 0 - not selected  1 - selected |
| 4  E04 | Which DiHA from the indication area hormones and metabolism have you **already prescribed** to your patients? [Multiple choice] | glucura Diabetestherapie  HelloBetter Diabetes und Depression  Mebix  My Dose Coach  Oviva Direkt für Adipositas  Una Health für Diabetes  Vitadio  Zanadio  Others | 0 - not selected  1 - selected |
| 5  E05 | Which DiHA from the indication area of hormones and metabolism do you **most frequently prescribe** to your patients? [Determine ranking] | glucura Diabetestherapie  HelloBetter Diabetes und Depression  Mebix  My Dose Coach  Oviva Direkt für Adipositas  Una Health für Diabetes  Vitadio  zanadio  Others | 1  2  3  4  5  6  7  8  9 |
| 6  E06 | To which age group do you **most frequently prescribe** a DiHA from the indication area of hormones and metabolism? [Determine ranking] | up to 25 years  26 to 35 years  36 to 45 years  46 to 55 years  56 to 65 years  over 65 years | 1  2  3  4  5  6 |
| 7  E07 | For which age group do you consider the prescription of a DiHA from the indication area of hormones and metabolism **to be useful**? [Multiple choice] | up to 25 years  26 to 35 years  36 to 45 years  46 to 55 years  56 to 65 years  over 65 years | 0 - not selected  1 - selected |
| 8  E08 | How often do you prescribe a DiHA from the indication area hormones and metabolism to your patients **on your own initiative**? | Never  Less than 1 x/month  Monthly  Weekly  Daily | 1  2  3  4  5  6 |
| 9  E09 | How often are you **asked by your patients** about the prescription of DiHA from the indication area hormones and metabolism? |  |  |
| 10  E10 | How often do you issue a **follow-up prescription** for a DiHA from the indication area hormones and metabolism? |  |  |
| 11  E11 | How often do you carry out an **evaluation of DiHA usage** with your patients? |  |  |
| 12  E12 | How high do you estimate the **demand for DiHA** from the indication area of hormones and metabolism? | Scale 0 (no need) to 10 (very high need) | None |
| 13  E13 | How likely do you think it is that you will **prescribe a DiHA from the indication area hormones and metabolism in the next 12 months?** | Very unlikely  Rather unlikely  Undecided  Rather likely  Very likely  I do not know | 1  2  3  4  5  6 |
| 14  E14 | Why didn't you prescribe DiHA to your patients? | Insufficient proof of efficacy  Lack of digital competence of patients  Lack of own digital competence  Little interest in prescribing DiHA  Insufficient experience  Fear of overuse or misuse  Insufficient connection to the practice software  Lack of time  Insufficient consideration of the additional time required for billing  Insufficient training opportunities  Data protection concerns if data is stored by third-party providers  Other issues | 0 - not selected  1 - selected |

### Healthcare effects

#### Potential healthcare effects

The following is an assessment of the **potential healthcare effects** that DiHA from the indication area of hormones and metabolism could **potentially achieve** in your opinion.

The use of DiHA from the indication area of hormones and metabolism could...

| No. | Question | Answer | Coding |
| --- | --- | --- | --- |
| 1  PV01 | ...prolong survival. | Do not agree at all  Do not agree  Undecided  Agree  Agree completely  I do not know | 1  2  3  4  5  6 |
| 2  PV02 | ...reduce discomfort and complications. |  |  |
| 3  PV03 | ...increase the quality of life. |  |  |
| 4  PV04 | ...improve joint decision-making between healthcare professional and patient. |  |  |
| 5  PV05 | ...increase the alignment of treatment with guidelines and recognized standards. |  |  |
| 6  PV06 | ...increase adherence to therapy. |  |  |
| 7  PV07 | ...improve access to hard-to-reach patient groups. |  |  |
| 8  PV08 | ...increase patient safety. |  |  |
| 9  PV09 | ...increase health literacy. |  |  |
| 10  PV10 | ...increase patient sovereignty. |  |  |
| 11  PV11 | ...improve disease management. |  |  |
| 12  PV12 | ...improve the involvement of relatives in the care process. |  |  |
| 13  PV13 | ...reduce HbA1c value. |  |  |
| 14  PV14 | ...reduce weight. |  |  |
| 15  PV15 | ...improve self-management. |  |  |
| 16  PV16 | What should be the **minimum reduction in the HbA1c value (in %)** of patients with diabetes mellitus for them to feel an improvement in their state of health? | Free text with % specification | None |
| 17  PV17 | What should be the **minimum weight reduction of overweight patients (BMI ≥ 25 kg/m2) (in %)** for patients to feel an improvement in their health? | Free text with % specification | None |

#### Healthcare effects actually observed

*If E01=yes and E02=yes*

In the following, we would like to ask you to assess the healthcare effects of DiHA from the indication area of hormones and metabolism that you believe **have already been achieved**.

The use of DiHA from the indication area hormones and metabolism has...

| No. | | Question | Answer | Coding |
| --- | --- | --- | --- | --- |
| 1  TV01 | ...prolonged survival. | | Do not agree at all  Do not agree  Undecided  Agree  Agree completely  I do not know | 1  2  3  4  5  6 |
| 2  TV02 | …reduced discomfort and complications. | |  |  |
| 3  TV03 | ...increased the quality of life. | |  |  |
| 4  TV04 | ...improved joint decision-making between healthcare professional and patient. | |  |  |
| 5  TV05 | ...increased the alignment of treatment with guidelines and recognized standards. | |  |  |
| 6  TV06 | ...increased adherence to therapy. | |  |  |
| 7  TV07 | ...improved access to hard-to-reach patient groups. | |  |  |
| 8  TV08 | ...increased patient safety. | |  |  |
| 9  TV09 | ...increased health literacy. | |  |  |
| 10  TV10 | ...increased patient sovereignty. | |  |  |
| 11  TV11 | ...improved disease management. | |  |  |
| 12  TV12 | ...improved the involvement of relatives in the care process. | |  |  |
| 13  TV13 | ...reduced HbA1c value. | |  |  |
| 14  TV14 | ...reduced weight. | |  |  |
| 15  TV15 | ...improved self-management. | |  |  |
| 16  TV16 | By what percentage did the **HbA1c value** of your patients with diabetes mellitus **decrease on average** as a result of using DiHA? | | Free text with % specification | None |
| 17  TV17 | By what percentage did the **weight** of your patients with overweight (BMI ≥ 25 kg/m2) **decrease on average** as a result of using DiHA? | | Free text with % specification | None |

### Barriers

The following is an assessment of **possible problems and barriers** for the prescription of DiHA from the indication area of hormones and metabolism.

In your opinion, which aspects pose the greatest problems and barriers for prescribing DiHA from the indication area of hormones and metabolism?

| No. | Question | | Answer | Coding |
| --- | --- | --- | --- | --- |
| 1  B01 | Lack of or insufficient evidence of benefit for patients | Do not agree at all  Do not agree  Undecided  Agree  Agree completely  I do not know | | 1  2  3  4  5  6 |
| 2  B02 | Application too complicated for patients |  |  |  |
| 3  B03 | Lack of technical support from the manufacturer |  |  |  |
| 4  B04 | Lack of patient motivation |  |  |  |
| 5  B05 | Uncertainty regarding the protection of privacy and the security of patients' personal (health) data |  |  |  |
| 6  B06 | Physician-patient relationship becomes more impersonal |  |  |  |
| 7  B07 | Lack of digital literacy among patients |  |  |  |
| 8  B08 | Insufficient adaptation of the DiHA to the individual needs of patients |  |  |  |
| 9  B09 | Poor integration or compatibility with existing practice software and tools |  |  |  |
| 10  B10 | Insufficient reimbursement of ancillary medical services, e.g. monitoring patient data and responding to queries |  |  |  |
| 11  B11 | Others *[Free text]* |  | | None |

### Study evidence

The following is an assessment of the **relevance of the study evidence** of DiHA from the indication area of hormones and metabolism.

If you decide to prescribe a DiHA from the indication area of hormones and metabolism, how important is it to you that ...

| No. | Question | Answer | Coding |
| --- | --- | --- | --- |
| 1  SE01 | ... a DiHA has already demonstrated a benefit and is permanently listed in the DiHA directory? | Unimportant  Rather unimportant  Partly, partly  Rather important  Important  I do not know | 1  2  3  4  5  6 |
| 2  SE02 | ... a DiHA - analogous to drug studies - could prove a medical benefit? |  |  |
| 3  SE03 | ... the proof of the positive healthcare effect was provided by a study with a high level of evidence (RCT)? |  |  |
| 4  SE04 | ... objective endpoints (BMI, weight, HbA1c value) were examined? |  |  |
| 5  SE05 | ... subjective endpoints (patient-reported outcomes, PROs) were examined? |  |  |
| 6  SE06 | ... clinically relevant, patient-relevant study results were achieved? |  |  |
| 7  SE07 | ... statistically significant study results were achieved? |  |  |

### Digital affinity

The following is an **assessment of your digital affinity**.

| No. | Question | Answer | Coding |
| --- | --- | --- | --- |
| DA01 | How digitally affine do you think you are? | Scale 0 (not at all digitally affine) to 10 (very digitally affine) | None |
| DA02 | Have you ever used a **health app** yourself as a patient? | Yes  No | 1  2 |
| DA03 | Have you ever used a **DiHA** yourself as a patient? | Yes  No | 1  2 |
| DA04 | Have you ever used a **manufacturer access** of a DiHA? | Yes  No | 1  2 |

### Soziodemographics

| No. | Question | Answer | Coding |
| --- | --- | --- | --- |
| 1 | Which **gender** do you identify with? | Male  Female  Diverse  Not specified | 1  2  3  4 |
| 2 | Please enter your **age**. | up to 25 years  26 to 35 years  36 to 45 years  46 to 55 years  56 to 65 years  over 65 years  Not specified | 1  2  3  4  5  6  7 |
| 3 | Do you have a **specialist title**? | Yes  No  Not specified | 1  2  3 |
| *#3 yes - continue with #4 to #6*  *#3 no - continue with #7* | | |  |
| 4 | In which **field** do you have a specialist title? [Multiple choice] | General medicine  Internal medicine  Pediatrics and adolescent medicine  Other [Free text]  Not specified | 0 - not selected  1 - selected |
| 5 | What is your **medical specialty**? Specialist for: [Multiple choice] | General medicine  Internal medicine without specialization  Internal medicine and angiology  Internal medicine and endocrinology and diabetology  Internal medicine and gastroenterology  Internal Medicine and Hematology and Oncology  Internal medicine and infectiology  Internal medicine and cardiology  Internal medicine and nephrology  Internal medicine and pneumology  Internal medicine and rheumatology  Pediatrics and adolescent medicine  Other [Free text]  Not specified | 0 - not selected  1 - selected |
| #4 Internal medicine or pediatrics and adolescent medicine - continue with #6  #4 everything else - continue with #7 | | |  |
| 6 | How do you work in terms of **health insurance fund medical care**? | Specialist care  General practitioner care  Not specified | 1  2  3 |
| 7 | Are you currently undergoing **further training** to become a specialist? | Yes  No  Not specified | 1  2  3 |
| *#7 yes - continue with #8 and #9*  *#7 no - continue with #10* | | |  |
| 8 | In which **field** are you currently undergoing further training to become a specialist? | General medicine  Internal medicine  Pediatrics and adolescent medicine  Other [Free text]  Not specified | 0 - not selected  1 - selected |
| 9 | Which specialist title do you hold after successfully completing your further training? **Specialist for:** | General medicine  Internal medicine without specialization  Internal medicine and angiology  Internal medicine and endocrinology and diabetology  Internal medicine and gastroenterology  Internal Medicine and Hematology and Oncology  Internal medicine and infectiology  Internal medicine and cardiology  Internal medicine and nephrology  Internal medicine and pneumology  Internal medicine and rheumatology  Pediatrics and adolescent medicine  Other [Free text]  Not specified | 0 - not selected  1 - selected |
| 10 | Do you have an **additional designation**? | Yes  No  Not specified | 1  2  3 |
| *#10 yes - continue with #11*  *#10 no - continue with #12* | | | |
| 11 | **Which additional title** do you have? [Multiple choice] | Diabetologist  Adiposiologist  Nutritional physician  Geriatrician  Other [Free text]  Not specified | 0 - not selected  1 - selected |
| 12 | How many **years of professional experience** do you have? | Less than 1 year  1-5 years  6-10 years  11-20 years  21-30 years  More than 30 years  Not specified | 1  2  3  4  5  6  7 |
| 13 | In which **federal state** do you work? | Baden-Wuerttemberg  Bavaria  Berlin  Brandenburg  Bremen  Hamburg  Hesse  Mecklenburg-Western Pomerania  Lower Saxony  North Rhine-Westphalia  Rhineland-Palatinate  Saarland  Saxony-Anhalt  Saxony-Anhalt  Schleswig-Holstein  Thuringia  Not specified | 1  2  3  4  5  6  7  8  9  10  11  12  13  14  15  16  17 |
| 14 | Where are you currently working? In a **municipality/city with ...** | Less than 5,000 inhabitants  5,000 to 20,000 inhabitants  20,001 to 100,000 inhabitants  100,001 to 500,000 inhabitants  More than 500,000 inhabitants  Not specified | 1  2  3  4  5  6 |
| 15 | Which **working model** best describes your field of activity? | Individual practice (without other colleagues)  Individual practice (with employed doctors)  Group practice  Medical care center  Hospital  Other [Free text] | 1  2  3  4  5  6 |
| 16 | How many **patients do you treat per quarter**? | Less than 500  500 to 750  751 to 1000  1001 to 1500  1501 to 2000  More than 2000  Not specified | 1  2  3  4  5  6  7 |

### Closing

Thank you very much for your participation, for your support of my dissertation and for your contribution to research!

The results of the survey will be published by the German Diabetes Society.

Melanie Mäder

melanie.maeder@uni-leipzig.de
