# Supplementary material for: Prescribing Experiences, Potentials, and Challenges of Digital Health Applications in the Field of Hormones and Metabolism: Cross-Sectional Survey Study of Health Care Providers in Germany
Source: JMIR Form Res. 2025 Dec 31;9:e77792. doi: 10.2196/77792 (PMC12805319; doi:10.2196/77792)
Supplement: Multimedia Appendix 2 [file formative_v9i1e77792_app2.docx]

# Multimedia Appendix 2: Distribution of the main socio-demographic characteristics of the HCPs

| Characteristics | | n | % |
| --- | --- | --- | --- |
|  | |  |  |
| Gender (n=290) |  |  |  |
|  | Male | 133 | 45.9 |
|  | Female | 154 | 53.1 |
|  | Diverse | 0 | 0 |
|  | Not specified | 3 | 1 |
| Age in years (n=290) |  |  |  |
|  | ≤ 25 | 0 | 0 |
|  | 26-35 | 16 | 5.5 |
|  | 36-45 | 72 | 24.8 |
|  | 46-55 | 86 | 29.7 |
|  | 56-65 | 82 | 28.3 |
|  | > 65 | 32 | 11 |
|  | Not specified | 2 | 0.7 |
| Specialization (n=290) |  |  |  |
|  | Yes | 278 | 95.9 |
|  | No | 11 | 3.8 |
|  | Not specified | 1 | 0.3 |
| Area of specialization (n=308)^A^ |  |  |  |
|  | General medicine | 47 | 15.3 |
|  | Internal medicine | 196 | 63.6 |
|  | Pediatrics and adolescent medicine | 37 | 12 |
|  | Other | 25 | 8.1 |
|  | Not specified | 3 | 1 |
| Type of specialist title (n=316)^A^ |  |  |  |
|  | General medicine | 43 | 13.6 |
|  | Internal medicine without specialization | 96 | 30.4 |
|  | Internal medicine and angiology | 4 | 1.3 |
|  | Internal medicine and endocrinology and diabetology | 64 | 20.3 |
|  | Internal medicine and gastroenterology | 12 | 3.8 |
|  | Internal Medicine and Hematology and Oncology | 0 | 0 |
|  | Internal medicine and infectiology | 0 | 0 |
|  | Internal medicine and cardiology | 12 | 3.8 |
|  | Internal medicine and nephrology | 8 | 2.5 |
|  | Internal medicine and pneumology | 2 | 0.6 |
|  | Internal medicine and rheumatology | 1 | 0.3 |
|  | Pediatrics and adolescent medicine | 37 | 11.7 |
|  | Other | 32 | 10.1 |
|  | Not specified | 5 | 1.6 |
| Activity within the framework of statutory health insurance care (n=233) |  |  |  |
|  | General practitioner care | 99 | 42.5 |
|  | Specialist care | 83 | 35.6 |
|  | Not specified | 51 | 21.9 |
| Additional title (n=289) |  |  |  |
|  | Yes | 238 | 82.4 |
|  | No | 48 | 16.6 |
|  | Not specified | 3 | 1 |
| Type of additional title (n=331)^A^ |  |  |  |
|  | Diabetologist | 211 | 63.8 |
|  | Adiposiologist | 20 | 6 |
|  | Nutritional physician | 42 | 12.7 |
|  | Geriatrician | 9 | 2.7 |
|  | Other | 47 | 14.2 |
|  | Not specified | 2 | 0.6 |
| Professional experience (n=290) |  |  |  |
|  | Less than 1 year | 0 | 0 |
|  | 1-5 years | 5 | 1.7 |
|  | 6-10 years | 31 | 10.7 |
|  | 11-20 years | 84 | 29 |
|  | 21-30 years | 84 | 29 |
|  | More than 30 years | 85 | 29.3 |
|  | Not specified | 1 | 0.3 |
| Federal state (n=290) |  |  |  |
|  | Baden-Wuerttemberg | 44 | 15.2 |
|  | Bavaria | 43 | 14.8 |
|  | Berlin | 16 | 5.5 |
|  | Brandenburg | 4 | 1.4 |
|  | Bremen | 2 | 0.7 |
|  | Hamburg | 10 | 3.4 |
|  | Hesse | 23 | 7.9 |
|  | Mecklenburg-Western Pomerania | 6 | 2.1 |
|  | Lower Saxony | 24 | 8.3 |
|  | North Rhine-Westphalia | 52 | 17.9 |
|  | Rhineland-Palatinate | 18 | 6.2 |
|  | Saarland | 4 | 1.4 |
|  | Saxony | 22 | 7.6 |
|  | Saxony-Anhalt | 6 | 2.1 |
|  | Schleswig-Holstein | 6 | 2.1 |
|  | Thuringia | 5 | 1.7 |
|  | Not specified | 5 | 1.7 |
| Activity in a municipality/city with (n=290) |  |  |  |
|  | Less than 5,000 inhabitants | 10 | 3.4 |
|  | 5,000 to 20,000 inhabitants | 62 | 21.4 |
|  | 20,001 to 100,000 inhabitants | 85 | 29.3 |
|  | 100,001 to 500,000 inhabitants | 57 | 19.7 |
|  | More than 500,000 inhabitants | 69 | 23.8 |
|  | Not specified | 7 | 2.4 |
| Working model (n=290) |  |  |  |
|  | Individual practice (without other colleagues) | 24 | 8.3 |
|  | Individual practice (with employed doctors) | 38 | 13.1 |
|  | Group practice | 80 | 27.6 |
|  | Medical care center | 37 | 12.8 |
|  | Hospital | 85 | 29.3 |
|  | Other | 16 | 5.5 |
|  | Not specified | 10 | 3.4 |
| Patients treated per quarter (n=290) |  |  |  |
|  | Less than 500 | 59 | 20.3 |
|  | 500 to 750 | 30 | 10.3 |
|  | 751 to 1000 | 51 | 17.6 |
|  | 1001 to 1500 | 49 | 16.9 |
|  | 1501 to 2000 | 35 | 12.1 |
|  | More than 2000 | 36 | 12.4 |
|  | Not specified | 30 | 10.3 |

^A^Multiple selections were possible
