# Supplementary material for: Prescribing Experiences, Potentials, and Challenges of Digital Health Applications in the Field of Hormones and Metabolism: Cross-Sectional Survey Study of Health Care Providers in Germany
Source: JMIR Form Res. 2025 Dec 31;9:e77792. doi: 10.2196/77792 (PMC12805319; doi:10.2196/77792)
Supplement: Multimedia Appendix 3 [file formative_v9i1e77792_app3.docx]

Multimedia Appendix 3: Experience with previous DiHA prescription in relation to sociodemographic variables, n (%)

| Item | Sociodemographics | Total | Yes | No | Statistic | Item | Sociodemographics | n | Yes | No | Statistic |
| --- | --- | --- | --- | --- | --- | --- | --- | --- | --- | --- | --- |
|  |  |  |  |  |  |  |  |  |  |  |  |
| *Already prescribed a DiHA* |  |  |  |  |  | *Already prescribed a DiHA from the indication area hormones and metabolism* |  |  |  |  |  |
|  |  | 350 (100) | 163 (46.6) | 187 (53.4) |  |  |  | 163 (100) | 139 (85.3) | 24 (14.7) |  |
| Gender |  |  |  |  |  | Gender |  |  |  |  |  |
|  | Male | 133 (45.9) | 53 (39) | 80 (51.9) | V=0.133 |  | Male | 53 (39) | 44 (37.9) | 9 (45) | V=0.069 |
|  | Female | 154 (53.1) | 81 (59.6) | 73 (47.4) | p=.077 |  | Female | 81 (59.6) | 70 (60.3) | 11 (55) | p=.724 |
|  | Diverse | 0 (0) | 0 (0) | 0 (0) | Fisher exact=.063 |  | Diverse | 0 (0) | 0 (0) | 0 (0) | Fisher exact=.728 |
|  | Not specified | 3 (1) | 2 (1.5) | 1 (0.6) |  |  | Not specified | 2 (1.5) | 2 (1.7) | 0 (0) |  |
| Age |  |  |  |  |  | Age |  |  |  |  |  |
|  | ≤ 25 | 0 (0) | 0 (0) | 0 (0) | V=0.178 |  | ≤ 25 | 0 (0) | 0 (0) | 0 (0) | V=0.152 |
|  | 26-35 | 16 (5.5) | 4 (2.9) | 12 (7.8) | p=.102 |  | 26-35 | 4 (2.9) | 4 (3.4) | 0 (0) | p=.676 |
|  | 36-45 | 72 (24.8) | 34 (25) | 38 (24.7) | Fisher exact=.092 |  | 36-45 | 34 (25) | 28 (24.1) | 6 (30) | Fisher exact=.633 |
|  | 46-55 | 86 (29.7) | 50 (36.8) | 36 (23.4) |  |  | 46-55 | 50 (36.8) | 45 (38.8) | 5 (25) |  |
|  | 56-65 | 82 (28.3) | 35 (25.7) | 47 (30.5) |  |  | 56-65 | 35 (25.7) | 29 (25) | 6 (30) |  |
|  | > 65 | 32 (11) | 12 (8.8) | 20 (13) |  |  | > 65 | 12 (8.8) | 9 (7.8) | 3 (15) |  |
|  | Not specified | 2 (0.7) | 1 (0.7) | 1 (0.6) |  |  | Not specified | 1 (0.7) | 1 (0.9) | 0 (0) |  |
| Specialization |  |  |  |  |  | Specialization |  |  |  |  |  |
|  | Yes | 278 (95.9) | 134 (98.5) | 144 (93.5) | V=0.128 |  | Yes | 134 (98.5) | 115 (99.1) | 1 (0.9) | V=0.122 |
|  | No | 11 (3.8) | 2 (1.5) | 9 (5.8) | p=.095 |  | No | 2 (1.5) | 1 (0.9) | 1 (0.9) | p=.156 |
|  | Not specified | 1 (0.3) | 0 (0) | 1 (0.6) | Fisher exact=.066 |  | Not specified | 0 (0) | 0 (0) | 0 (0) | Fisher exact=.273 |
| Activity within the framework of statutory health insurance care |  |  |  |  |  | Activity within the framework of statutory health insurance care |  |  |  |  |  |
|  | General practitioner care | 99 (42.5) | 70 (66) | 29 (22.8) | V=0.481 |  | General practitioner care | 70 (66) | 62 (66.7) | 8 (61.5) | V=0.190 |
|  | Specialist care | 83 (35.6) | 31 (29.2) | 52 (40.9) | p<.001 |  | Specialist care | 31 (29.2) | 28 (30.1) | 3 (23.1) | p=.149 |
|  | Not specified | 51 (21.9) | 5 (4.7) | 46 (36.2) | Fisher exact<.001 |  | Not specified | 5 (4.7) | 3 (3.2) | 2 (15.4) | Fisher exact=.181 |
| Additional title |  |  |  |  |  | Additional title |  |  |  |  |  |
|  | Yes | 238 (82.4) | 119 (88.1) | 119 (77.3) | V=0.142 |  | Yes | 119 (88.1) | 103 (89.6) | 16 (80) | V=0.215 |
|  | No | 48 (16.6) | 15 (11.1) | 33 (21.4) | p=.053 |  | No | 15 (11.1) | 12 (10.4) | 3 (15) | p=.044 |
|  | Not specified | 3 (1) | 1 (0.7) | 2 (1.3) | Fisher exact=.043 |  | Not specified | 1 (0.7) | 0 (0) | 1 (5) | Fisher exact=.112 |
| Professional experience |  |  |  |  |  | Professional experience |  |  |  |  |  |
|  | Less than 1 year | 0 (0) | 0 (0) | 0 (0) | V=0.128 |  | Less than 1 year | 0 (0) | 0 (0) | 0 (0) | V=0.196 |
|  | 1-5 years | 5 (1.7) | 2 (1.5) | 3 (1.9) | p=.446 |  | 1-5 years | 2 (1.5) | 1 (0.9) | 1 (5) | p=.264 |
|  | 6-10 years | 31 (10.7) | 12 (8.8) | 19 (12.3) | Fisher exact=.446 |  | 6-10 years | 12 (8.8) | 10 (8.6) | 2 (10) | Fisher exact=.240 |
|  | 11-20 years | 84 (29) | 42 (30.9) | 42 (27.3) |  |  | 11-20 years | 42 (30.9) | 38 (32.8) | 4 (20) |  |
|  | 21-30 years | 84 (29) | 45 (33.1) | 39 (25.3) |  |  | 21-30 years | 45 (33.1) | 40 (34.5) | 5 (25) |  |
|  | More than 30 years | 85 (29.3) | 35 (25.7) | 50 (32.5) |  |  | More than 30 years | 35 (25.7) | 27 (23.3) | 8 (40) |  |
|  | Not specified | 1 (0.3) | 0 (0) | 1 (0.6) |  |  | Not specified | 0 (0) | 0 (0) | 0 (0) |  |
| Federal state |  |  |  |  |  | Federal state |  |  |  |  |  |
|  | Baden-Wuerttemberg | 44 (15.2) | 22 (16.2) | 22 (14.3) | V=0.165 |  | Baden-Wuerttemberg | 22 (16.2) | 21 (18.1) | 1 (5) | V=0.353 |
|  | Bavaria | 43 (14.8) | 19 (14) | 24 (15.6) | p=.951 |  | Bavaria | 19 (14) | 12 (10.3) | 7 (35) | p=.388 |
|  | Berlin | 16 (5.5) | 7 (5.1) | 9 (5.8) | Fisher exact=.943 |  | Berlin | 7 (5.1) | 7 (6) | 0 (0) | Fisher exact=.379 |
|  | Brandenburg | 4 (1.4) | 2 (1.5) | 2 (1.3) |  |  | Brandenburg | 2 (1.5) | 1 (0.9) | 1 (5) |  |
|  | Bremen | 2 (0.7) | 1 (0.7) | 2 (1.3) |  |  | Bremen | 1 (0.7) | 1 (0.9) | 0 (0) |  |
|  | Hamburg | 10 (3.4) | 5 (3.7) | 5 (3.2) |  |  | Hamburg | 5 (3.7) | 4 (3.4) | 1 (5) |  |
|  | Hesse | 23 (7.9) | 11 (8.1) | 12 (7.8) |  |  | Hesse | 11 (8.1) | 9 (7.8) | 2 (10) |  |
|  | Mecklenburg-Western Pomerania | 6 (2.1) | 3 (2.2) | 3 (1.9) |  |  | Mecklenburg-Western Pomerania | 3 (2.2) | 3 (2.6) | 0 (0) |  |
|  | Lower Saxony | 24 (8.3) | 11 (8.1) | 13 (8.4) |  |  | Lower Saxony | 11 (8.1) | 10 (8.6) | 1 (5) |  |
|  | North Rhine-Westphalia | 52 (17.9) | 19 (14) | 33 (21.4) |  |  | North Rhine-Westphalia | 19 (14) | 16 (13.8) | 3 (15) |  |
|  | Rhineland-Palatinate | 18 (6.2) | 8 (5.9) | 10 (6.5) |  |  | Rhineland-Palatinate | 8 (5.9) | 6 (5.2) | 2 (10) |  |
|  | Saarland | 4 (1.4) | 2 (1.5) | 2 (1.3) |  |  | Saarland | 2 (1.5) | 2 (1.7) | 0 (0) |  |
|  | Saxony | 22 (7.6) | 15 (11) | 7 (4.5) |  |  | Saxony | 15 (11) | 14 (12.1) | 1 (5) |  |
|  | Saxony-Anhalt | 6 (2.1) | 4 (2.9) | 2 (1.3) |  |  | Saxony-Anhalt | 4 (2.9) | 3 (2.6) | 1 (5) |  |
|  | Schleswig-Holstein | 6 (2.1) | 3 (2.2) | 3 (1.9) |  |  | Schleswig-Holstein | 3 (2.2) | 3 (2.6) | 1 (5) |  |
|  | Thuringia | 5 1.7) | 2 (1.5) | 3 (1.9) |  |  | Thuringia | 2 (1.5) | 2 (1.7) | 0 (0) |  |
|  | Not specified | 5 (1.7) | 2 (1.5) | 3 (1.9) |  |  | Not specified | 2 (1.5) | 2 (1.7) | 0 (0) |  |
| Activity in a municipality/city with |  |  |  |  |  | Activity in a municipality/city with |  |  |  |  |  |
|  | Less than 5,000 inhabitants | 10 (3.4) | 6 (4.4) | 4 (2.6) | V=0.231 |  | Less than 5,000 inhabitants | 6 (4.4) | 5 (4.3) | 1 (5) | V=0.133 |
|  | 5,000 to 20,000 inhabitants | 62 (21.4) | 36 (26.5) | 26 (16.9) | p=.009 |  | 5,000 to 20,000 inhabitants | 36 (26.5) | 28 (24.1) | 8 (40) | p=.661 |
|  | 20,001 to 100,000 inhabitants | 85 (29.3) | 30 (22.1) | 55 (35.7) | Fisher exact=.007 |  | 20,001 to 100,000 inhabitants | 30 (22.1) | 26 (22.4) | 4 (20) | Fisher exact=.662 |
|  | 100,001 to 500,000 inhabitants | 57 (19.7) | 28 (20.6) | 29 (18.8) |  |  | 100,001 to 500,000 inhabitants | 28 (20.6) | 25 (21.6) | 3 (15) |  |
|  | More than 500,000 inhabitants | 69 (23.8) | 36 (26.5) | 33 (21.4) |  |  | More than 500,000 inhabitants | 36 (26.5) | 32 (27.6) | 4 (20) |  |
|  | Not specified | 7 (2.4) | 0 (0) | 7 (4.5) |  |  | Not specified | 0 (0) | 0 (0) | 0 (0) |  |
| Working model |  |  |  |  |  | Working model |  |  |  |  |  |
|  | Individual practice (without other colleagues) | 24 (8.3) | 16 (11.8) | 8 (5.2) | V=0.437 |  | Individual practice (without other colleagues) | 16 (11.8) | 12 (10.3) | 4 (20) | V=0.151 |
|  | Individual practice (with employed doctors) | 38 (13.1) | 24 (17.6) | 14 (9.1) | p<.001 |  | Individual practice (with employed doctors) | 24 (17.6) | 22 (19) | 2 (10) | p=.795 |
|  | Group practice | 80 (27.6) | 54 (39.7) | 26 (16.9) | Fisher exact<.001 |  | Group practice | 54 (39.7) | 46 (39.7) | 8 (40) | Fisher exact=.671 |
|  | Medical care center | 37 (12.8) | 20 (14.7) | 17 (11) |  |  | Medical care center | 20 (14.7) | 18 (15.5) | 2 (10) |  |
|  | Hospital | 85 (29.3) | 17 (12.5) | 68 (44.2) |  |  | Hospital | 17 (12.5) | 14 (12.1) | 3 (15) |  |
|  | Other | 16 (5.5) | 4 (2.9) | 12 (7.8) |  |  | Other | 4 (2.9) | 3 (2.6) | 1 (5) |  |
|  | Not specified | 10 (3.4) | 1 (0.7) | 9 (5.8) |  |  | Not specified | 1 (0.7) | 1 (0.9) | 0 (0) |  |
| Patients treated per quarter |  |  |  |  |  | Patients treated per quarter |  |  |  |  |  |
|  | Less than 500 | 59 (20.3) | 9 (6.6) | 50 (32.5) | V=0.477 |  | Less than 500 | 9 (6.6) | 7 (6) | 2 (10) | V=0.151 |
|  | 500 to 750 | 30 (10.3) | 12 (8.8) | 18 (11.7) | p<.001 |  | 500 to 750 | 12 (8.8) | 12 (10.3) | 0 (0) | p=.795 |
|  | 751 to 1000 | 51 (17.6) | 30 (22.1) | 21 (13.6) | Fisher exact<.001 |  | 751 to 1000 | 30 (22.1) | 25 (21.6) | 5 (25) | Fisher exact=.761 |
|  | 1001 to 1500 | 49 16.9) | 33 (24.3) | 16 (10.4) |  |  | 1001 to 1500 | 33 (24.3) | 29 (25) | 4 (20) |  |
|  | 1501 to 2000 | 35 (12.1) | 23 (16.9) | 12 (7.8) |  |  | 1501 to 2000 | 23 (16.9) | 19 (16.4) | 4 (20) |  |
|  | More than 2000 | 36 (12.4) | 26 (19.1) | 10 (6.5) |  |  | More than 2000 | 26 (19.1) | 22 (19) | 4 (20) |  |
|  | Not specified | 30 (10.3) | 3 (2.2) | 27 (17.5) |  |  | Not specified | 3 (2.2) | 2 (1.7) | 1 (5) |  |
| Ever used a health app as a patient |  |  |  |  |  | Ever used a health app as a patient |  |  |  |  |  |
|  | Yes | 142 (48.6) | 66 (48.2) | 76 (49) | V=0.009 |  | Yes | 66 (48.2) | 55 (47) | 11 (55) | V=0.056 |
|  | No | 150 (51.4) | 71 (51.8) | 79 (51) | p=.884 |  | No | 71 (51.8) | 62 (53) | 9 (45) | p=.509 |
|  |  |  |  |  | Fisher exact=.907 |  |  |  |  |  | Fisher exact=.630 |
| Ever used a DiHA as a patient |  |  |  |  |  | Ever used a DiHA as a patient |  |  |  |  |  |
|  | Yes | 39 (13.4) | 20 (14.6) | 19 (12.3) | V=0.034 |  | Yes | 20 (14.6) | 15 (12.8) | 5 (25) | V=0.122 |
|  | No | 253 (86.6) | 117 (85.4) | 136 (87.7) | p=.557 |  | No | 117 (85.4) | 102 (87.2) | 15 (75) | p=.154 |
|  |  |  |  |  | Fisher exact=.607 |  |  |  |  |  | Fisher exact=.173 |
| Ever used a DiHA manufacturer access |  |  |  |  |  | Ever used a DiHA manufacturer access |  |  |  |  |  |
|  | Yes | 101 (34.6) | 75 (54.7) | 26 (16.8) | V=0.398 |  | Yes | 75 (54.7) | 72 (61.5) | 3 (15) | V=0.330 |
|  | No | 191 (65.4) | 62 (45.3) | 129 (83.2) | p<.001 |  | No | 62 (45.3) | 45 (38.5) | 17 (85) | p<.001 |
|  |  |  |  |  | Fisher exact<.001 |  |  |  |  |  | Fisher exact<.001 |

p=p-value | V=Cramér’s V

The descriptive statistics are to be interpreted within the variable “Experience with previous DiHA prescription”.
