# Supplementary material for: Prescribing Experiences, Potentials, and Challenges of Digital Health Applications in the Field of Hormones and Metabolism: Cross-Sectional Survey Study of Health Care Providers in Germany
Source: JMIR Form Res. 2025 Dec 31;9:e77792. doi: 10.2196/77792 (PMC12805319; doi:10.2196/77792)
Supplement: Multimedia Appendix 4 [file formative_v9i1e77792_app4.docx]

Multimedia Appendix 4: Prescription intention in relation to sociodemographic variables, n (%)

|  | Soziodemographics | Total | Very unlikely | Rather unlikely | Undecided | More likely | Very likely | I don’t know | Statistic |
| --- | --- | --- | --- | --- | --- | --- | --- | --- | --- |
|  |  |  |  |  |  |  |  |  |  |
| *Will precribe DiHA^A^ in the next 12 months* |  |  |  |  |  |  |  |  |  |
|  |  | 348 (100) | 83 (23.9) | 66 (19) | 44 (12.6) | 61 (17.5) | 80 (23) | 14 (4) |  |
| Gender |  |  |  |  |  |  |  |  |  |
|  | Male | 133 (45.9) | 32 (47.5) | 27 (50) | 19 (51.4) | 24 (43.6) | 24 (35.8) | 7 (70) | V=0.137 |
|  | Female | 154 (53.1) | 33 (49.3) | 27 (50) | 18 (48.6) | 30 (54.5) | 43 (64.2) | 3 (30) | p=.371 |
|  | Diverse | 0 (0) | 0 (0) | 0 (0) | 0 (0) | 0 (0) | 0 (0) | 0 (0) | Fisher exact=.368 |
|  | Not specified | 3 (1) | 2 (3) | 0 (0) | 0 (0) | 1 (1.8) | 0 (0) | 0 (0) |  |
| Age |  |  |  |  |  |  |  |  |  |
|  | ≤ 25 | 0 (0) | 0 (0) | 0 (0) | 0 (0) | 0 (0) | 0 (0) | 0 (0) | ρ=-0.036 |
|  | 26-35 | 16 (5.5) | 4 (6) | 4 (7.4) | 1 (2.7) | 1 (1.8) | 6 (9) | 0 (0) | p=.540 |
|  | 36-45 | 72 (24.8) | 17 (25) | 9 (16.7) | 13 (35.1) | 13 (23.6) | 18 (26.9) | 2 (20) |  |
|  | 46-55 | 86 (29.7) | 19 (28) | 14 (25.9) | 10 (27) | 15 (27.3) | 26 (38.8) | 2 (20) |  |
|  | 56-65 | 82 (28.3) | 21 (31.3) | 20 (37) | 11 (29.7) | 14 (25) | 14 (20.9) | 2 (20) |  |
|  | > 65 | 32 (11) | 5 (7.5) | 7 (13) | 2 (5.4) | 11 (20) | 3 (4.5) | 4 (40) |  |
|  | Not specified | 2 (0.7) | 1 (1.5) | 0 (0) | 0 (0) | 1 (1.8) | 0 (0) | 0 (0) |  |
| Specialization |  |  |  |  |  |  |  |  |  |
|  | Yes | 278 (95.9) | 64 (95.5) | 54 (100) | 33 (89.2) | 53 (96.4) | 65 (97) | 9 (90) | V=0.142 |
|  | No | 11 (3.8) | 2 (3) | 0 (0) | 4 (10.8) | 2 (3.6) | 2 (3) | 1 (10) | p=.302 |
|  | Not specified | 1 (0.3) | 1 (1.5) | 0 (0) | 0 (0) | 0 (0) | 0 (0) | 0 (0) | Fisher exact=.218 |
| Activity within the framework of statutory health insurance care |  |  |  |  |  |  |  |  |  |
|  | General practitioner care | 99 (42.5) | 14 (26.9) | 18 (37.5) | 12 (46.2) | 17 (37.8) | 38 (67.9) | 0 (0) | V=0.266 |
|  | Specialist care | 83 (35.6) | 19 (36.5) | 17 (35.4) | 8 (30.8) | 22 (48.9) | 13 (23.2) | 4 (66.7) | p<.001 |
|  | Not specified | 51 (21.9) | 19 (36.5) | 13 (27.1) | 6 (23.1) | 6 (13.3) | 5 (8.9) | 2 (33.3) | Fisher exact<.001 |
| Additional title |  |  |  |  |  |  |  |  |  |
|  | Yes | 238 (82.4) | 57 (85.1) | 46 (85.2) | 30 (81.1) | 43 (78.2) | 54 (81.8) | 8 (80) | V=0.093 |
|  | No | 48 (16.6) | 9 (13.4) | 7 (13) | 6 (16.2) | 12 (21.8) | 12 (18.2) | 2 (20) | p=.893 |
|  | Not specified | 3 (1) | 1 (1.) | 1 (1.9) | 1 (2.7) | 0 (0) | 0 (0) | 0 (0) | Fisher exact=.896 |
| Professional experience |  |  |  |  |  |  |  |  |  |
|  | Less than 1 year | 0 (0) | 0 (0) | 0 (0) | 0 (0) | 0 (0) | 0 (0) | 0 (0) | ρ=-0.072 |
|  | 1-5 years | 5 (1.7) | 0 (0) | 1 (1.9) | 1 (2.7) | 1 (1.8) | 2 (3) | 0 (0) | p=.219 |
|  | 6-10 years | 31 (10.7) | 6 (9) | 6 (11.1) | 3 (8.1) | 5 (9.1) | 11 (16.4) | 0 (0) |  |
|  | 11-20 years | 84 (29) | 24 (35.8) | 8 (14.8) | 14 (37.8) | 16 (29.1) | 19 (28.4) | 3 (30) |  |
|  | 21-30 years | 84 (29) | 17 (25.4) | 16 (29.6) | 10 (27) | 16 (29.1) | 23 (34.3) | 2 (20) |  |
|  | More than 30 years | 85 (29.3) | 19 (28.4) | 23 (42.6) | 9 (24.3) | 17 (30.9) | 12 (17.9) | 5 (50) |  |
|  | Not specified | 1 (0.3) | 1 (1.5) | 0 (0) | 0 (0) | 0 (0) | 0 (0) | 0 (0) |  |
| Federal state |  |  |  |  |  |  |  |  |  |
|  | Baden-Wuerttemberg | 44 (15.2) | 7 (10.4) | 6 (11.1) | 7 (18.9) | 9 (16.4) | 14 (20.9) | 1 (10) | V=0.236 |
|  | Bavaria | 43 (14.8) | 18 (26.9) | 6 (11.1) | 6 (16.2) | 5 (9.1) | 7 (10.4) | 1 (10) | p=.455 |
|  | Berlin | 16 (5.5) | 4 (6) | 2 (3.7) | 4 (10.8) | 2 (3.6) | 4 (6) | 0 (0) |  |
|  | Brandenburg | 4 (1.4) | 1 (1.5) | 1 (1.9) | 0 (0) | 2 (3.6) | 0 (0) | 0 (0) |  |
|  | Bremen | 2 (0.7) | 2 (3) | 0 (0) | 0 (0) | 0 (0) | 0 (0) | 0 (0) |  |
|  | Hamburg | 10 (3.4) | 2 (3) | 2 (3.7) | 2 (5.4) | 2 (3.6) | 2 (3) | 0 (0) |  |
|  | Hesse | 23 (7.9) | 6 (9) | 3 (5.6) | 4 (10.8) | 5 (9.1) | 5 (7.5) | 0 (0) |  |
|  | Mecklenburg-Western Pomerania | 6 (2.1) | 1 (1.5) | 2 (3.7) | 0 (0) | 1 (1.8) | 1 (1.5) | 1 (10) |  |
|  | Lower Saxony | 24 (8.3) | 5 (7.5) | 5 (9.3) | 0 (0) | 4 (7.3) | 9 (13.4) | 1 (10) |  |
|  | North Rhine-Westphalia | 52 (17.9) | 9 (13.4) | 12 (22.2) | 9 (24.3) | 9 (16.4) | 8 (11.9) | 5 (50) |  |
|  | Rhineland-Palatinate | 18 (6.2) | 5 (7.5) | 3 (5.6) | 2 (5.4) | 6 (10.9) | 2 (3) | 0 (0) |  |
|  | Saarland | 4 (1.4) | 1 (1.5) | 1 (1.9) | 0 (0) | 2 (3.6) | 0 (0) | 0 (0) |  |
|  | Saxony | 22 (7.6) | 3 (4.5) | 5 (9.3) | 2 (5.4) | 3 (5.5) | 9 (13.4) | 0 (0) |  |
|  | Saxony-Anhalt | 6 (2.1) | 0 (0) | 3 (5.6) | 0 (0) | 2 (3.6) | 1 (1.5) | 0 (0) |  |
|  | Schleswig-Holstein | 6 (2.1) | 1 (1.5) | 0 (0) | 0 (0) | 1 (1.8) | 3 (4.5) | 1 (10) |  |
|  | Thuringia | 5 (1.7) | 0 (0) | 1 (1.9) | 1 (2.7) | 1 (1.8) | 2 (3) | 0 (0) |  |
|  | Not specified | 5 (1.7) | 2 (3) | 2 (3.7) | 0 (0) | 1 (1.8) | 0 (0) | 0 (0) |  |
| Activity in a municipality/city with |  |  |  |  |  |  |  |  |  |
|  | Less than 5,000 inhabitants | 10 (3.4) | 4 (6) | 0 (0) | 0 (0) | 2 (3.6) | 3 (4.5) | 1 (10) | ρ=0.011 |
|  | 5,000 to 20,000 inhabitants | 62 (21.4) | 16 (23.9) | 7 (13) | 9 (24.3) | 13 (23.6) | 15 (22.4) | 2 (20) | p=.851 |
|  | 20,001 to 100,000 inhabitants | 85 (29.3) | 19 (28.4) | 22 (40.7) | 14 (37.8) | 12 (21.8) | 16 (23.9) | 2 (20) |  |
|  | 100,001 to 500,000 inhabitants | 57 (19.7) | 11 (16.4) | 10 (18.5) | 2 (5.4) | 16 (29.1) | 15 (22.4) | 3 (30) |  |
|  | More than 500,000 inhabitants | 69 (23.8) | 15 (22.4) | 13 (24.1) | 12 (32.4) | 11 (20) | 17 (25.4) | 1 (10) |  |
|  | Not specified | 7 (2.4) | 2 (3) | 2 (3.7) | 0 (0) | 1 (1.8) | 1 (1.5) | 1 (10) |  |
| Working model |  |  |  |  |  |  |  |  |  |
|  | Individual practice (without other colleagues) | 24 (8.3) | 7 (10.4) | 4 (7.4) | 4 (10.8) | 3 (5.5) | 5 (7.5) | 1 (10) | V=0.246 |
|  | Individual practice (with employed doctors) | 38 (13.1) | 7 (10.4) | 8 (14.8) | 3 (8.1) | 9 (16.4) | 10 (14.9) | 1 (10) | p<.001 |
|  | Group practice | 80 (27.6) | 12 (17.9) | 12 (22.2) | 8 (21.6) | 22 (40) | 25 (37.3) | 1 (10) | Fisher exact<.001 |
|  | Medical care center | 37 (12.8) | 3 (4.5) | 7 (13) | 7 (18.9) | 6 (10.9) | 14 (20.9) | 0 (0) |  |
|  | Hospital | 85 (29.3) | 27 (40.3) | 20 (37) | 12 (32.4) | 14 (25.5) | 11 (16.4) | 1 (10) |  |
|  | Other | 16 (5.5) | 8 (11.9) | 1 (1.9) | 2 (5.4) | 1 (1.8) | 2 (3) | 2 (20) |  |
|  | Not specified | 10 (3.4) | 3 (4.5) | 2 (3.7) | 1 (2.7) | 0 (0) | 0 (0) | 4 (40) |  |
| Patients treated per quarter |  |  |  |  |  |  |  |  | ρ=0.085 |
|  | Less than 500 | 59 (20.3) | 18 (26.9) | 13 (24.1) | 14 (37.8) | 5 (9.1) | 8 (11.9) | 1 (10) | p=.147 |
|  | 500 to 750 | 30 (10.3) | 6 (9) | 6 (11.1) | 4 (10.8) | 3 (5.5) | 10 (14.9) | 1 (10) |  |
|  | 751 to 1000 | 51 (17.6) | 10 (14.9) | 8 (14.8) | 6 (16.2) | 15 (27.3) | 10 (14.9) | 2 (20) |  |
|  | 1001 to 1500 | 49 (16.9) | 7 (10.4) | 13 (24.1) | 4 (10.8) | 8 (14.5) | 17 (25.4) | 0 (0) |  |
|  | 1501 to 2000 | 35 (12.1) | 7 (10.4) | 5 (9.3) | 5 (13.5) | 5 (9.1) | 13 (19.4) | 0 (0) |  |
|  | More than 2000 | 36 (12.4) | 6 (9) | 5 (9.3) | 4 (10.8) | 13 (23.6) | 6 (9) | 2 (20) |  |
|  | Not specified | 13 (19.4) | 4 (7.4) | 0 (0) | 6 (10.9) | 3 (4.5) | 4 (40) | 30 (10.3) |  |
| Ever used a health app as a patient |  |  |  |  |  |  |  |  |  |
|  | Yes | 142 (48.6) | 25 (36.8) | 26 (48.1) | 22 (57.9) | 25 (45.5) | 38 (56.7) | 6 (60) | V=0.162 |
|  | No | 150 (51.4) | 43 (63.2) | 28 (51.9) | 16 (42.1) | 30 (54.5) | 29 (43.3) | 4 (40) | p=.177 |
|  |  |  |  |  |  |  |  |  | Fisher exact=.179 |
| Ever used a DiHA as a patient |  |  |  |  |  |  |  |  |  |
|  | Yes | 39 (13.4) | 8 (11.8) | 4 (7.4) | 8 (21.1) | 5 (9.1) | 10 (14.9) | 4 (40) | V=0.193 |
|  | No | 253 (86.6) | 60 (88.2) | 50 (92.6) | 30 (78.9) | 50 (90.9) | 57 (85.1) | 6 (60) | p=.054 |
|  |  |  |  |  |  |  |  |  | Fisher exact=.052 |
| Ever used a DiHA manufacturer access |  |  |  |  |  |  |  |  |  |
|  | Yes | 101 (34.6) | 12 (17.6) | 17 (31.5) | 9 (23.7) | 17 (30.9) | 41 (61.2) | 5 (50) | V=0.337 |
|  | No | 191 (65.4) | 56 (82.4) | 37 (68.5) | 29 (76.3) | 38 (69.1) | 26 (38.8) | 5 (50) | p<.001 |
|  |  |  |  |  |  |  |  |  | Fisher exact<.001 |

^A^ from the indication area hormones and metabolism

ρ=Spearman’s rank correlation coefficient | p=p-value | V=Cramér’s V

The descriptive statistics are to be interpreted within the variable “Prescription intention”
