# Supplementary figures and images for: Prescribing Experiences, Potentials, and Challenges of Digital Health Applications in the Field of Hormones and Metabolism: Cross-Sectional Survey Study of Health Care Providers in Germany
Source: JMIR Form Res. 2025 Dec 31;9:e77792. doi: 10.2196/77792 (PMC12805319; doi:10.2196/77792)

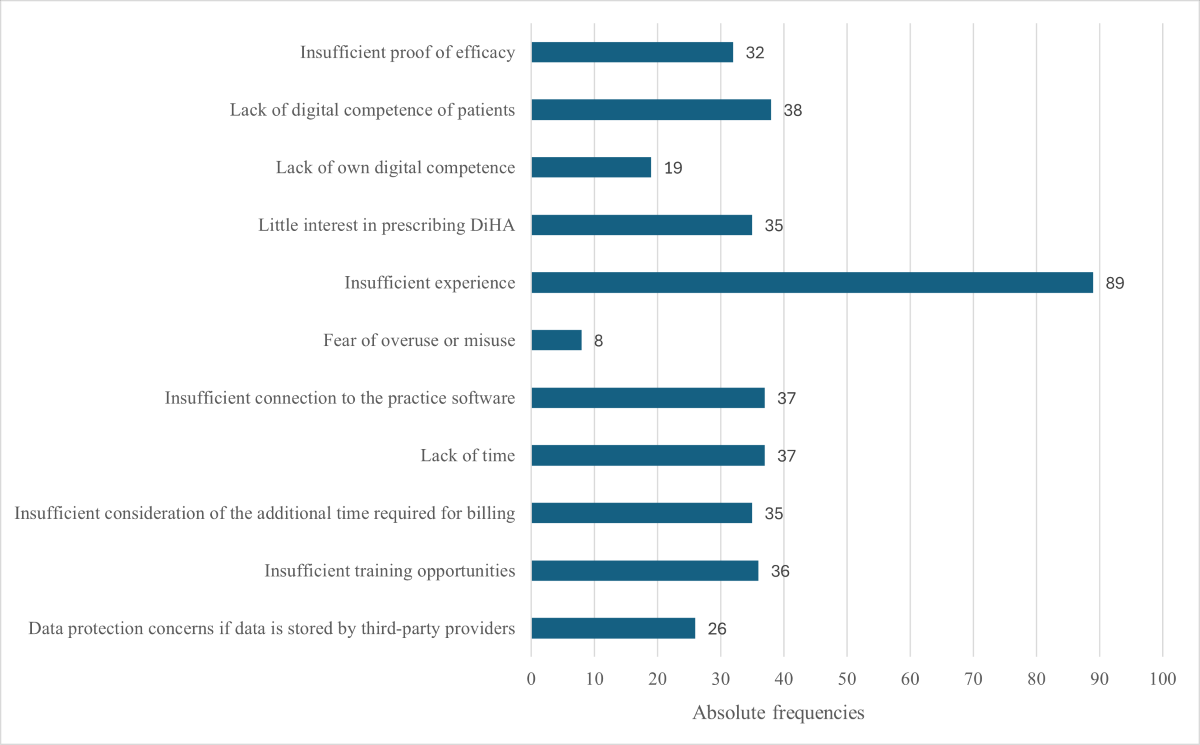

Supplement: Multimedia Appendix 5 [file formative_v9i1e77792_app5.png]
