# Supplementary material for: Prescribing Experiences, Potentials, and Challenges of Digital Health Applications in the Field of Hormones and Metabolism: Cross-Sectional Survey Study of Health Care Providers in Germany
Source: JMIR Form Res. 2025 Dec 31;9:e77792. doi: 10.2196/77792 (PMC12805319; doi:10.2196/77792)
Supplement: Multimedia Appendix 7 [file formative_v9i1e77792_app7.docx]

Multimedia Appendix 7: Prescription frequency in relation to sociodemographic variables, n (%)

|  | Soziodemographics | Total | Never | Less than 1 x/month | Monthly | Weekly | Daily | Statistic |
| --- | --- | --- | --- | --- | --- | --- | --- | --- |
|  |  |  |  |  |  |  |  |  |
| *Prescribing DiHA^A^ on own initiative* |  |  |  |  |  |  |  |  |
|  |  | 139 (100) | 9 (6.5) | 75 (54.) | 40 (28.8) | 11 (7.9) | 4 (2.9) |  |
| Gender |  |  |  |  |  |  |  |  |
|  | Male | 44 (37.9) | 0 (0) | 26 (40.6) | 14 (42.4) | 4 (44.4) | 0 (0) | V=0.237 |
|  | Female | 70 (60.3) | 6 (85.7) | 37 (57.8) | 19 (57.6) | 5 (55.6) | 3 (100) | p=.110 |
|  | Diverse | 0 (0) | 0 (0) | 0 (0) | 0 (0) | 0 (0) | 0 (0) | Fisher exact=.124 |
|  | Not specified | 2 (1.7) | 1 (14.3) | 1 (1.6) | 0 (0) | 0 (0) | 0 (0) |  |
| Age |  |  |  |  |  |  |  |  |
|  | ≤ 25 | 0 (0) | 0 (0) | 0 (0) | 0 (0) | 0 (0) | 0 (0) | ρ=-0.058 |
|  | 26-35 | 4 (3.4) | 0 (0) | 1 (1.6) | 3 (9.1) | 0 (0) | 0 (0) | p=.537 |
|  | 36-45 | 28 (24.1) | 3 (42.9) | 16 (25) | 5 (15.2) | 3 (33.3) | 1 (33.3) |  |
|  | 46-55 | 45 (38.8) | 2 (28.6) | 22 (34.4) | 16 (48.5) | 4 (44.4) | 1 (33.3) |  |
|  | 56-65 | 29 (25) | 2 (28.6) | 18 (28.1) | 6 (18.2) | 2 (22.2) | 1 (33.3) |  |
|  | > 65 | 9 (7.8) | 0 (0) | 6 (9.4) | 3 (9.1) | 0 (0) | 0 (0) |  |
|  | Not specified | 1 (0.9) | 0 (0) | 1 (1.6) | 0 (0) | 0 (0) | 0 (0) |  |
| Specialization |  |  |  |  |  |  |  |  |
|  | Yes | 115 (99.1) | 7 (100) | 63 (98.4) | 33 (100) | 9 (100) | 3 (100) | V=0.084 |
|  | No | 0 1 (0.9) | 1 (1.6) | 0 (0) | 0 (0) | 0 (0) | 0 (0) | p=.936 |
|  | Not specified | 0 (0) | 0 (0) | 0 (0) | 0 (0) | 0 (0) | 0 (0) |  |
| Activity within the framework of statutory health insurance care |  |  |  |  |  |  |  |  |
|  | General practitioner care | 62 (66.7) | 3 (60) | 35 (68.6) | 16 (59.3) | 6 (85.7) | 2 (66.7) | V=0.168 |
|  | Specialist care | 28 (30.1) | 2 (40) | 13 (25.5) | 11 (40.7) | 1 (14.3) | 1 (33.3) | p=.729 |
|  | Not specified | 3 (3.2) | 0 (0) | 3 (5.9) | 0 (0) | 0 (0) | 0 (0) | Fisher exact=.690 |
| Additional title |  |  |  |  |  |  |  |  |
|  | Yes | 103 (89.6) | 6 (85.7) | 59 (92.2) | 26 (81.3) | 9 (100) | 3 (100) | V=0.194 |
|  | No | 12 (10.4) | 1 (14.3) | 5 (7.8) | 6 (18.8) | 0 (0) | 0 (0) | p=.361 |
|  | Not specified | 0 (0.00) |  |  |  |  |  | Fisher exact=.317 |
| Professional experience |  |  |  |  |  |  |  |  |
|  | Less than 1 year | 0 (0) | 0 (0) | 0 (0) | 0 (0) | 0 (0) | 0 (0) | ρ=0.011 |
|  | 1-5 years | 1 (0.9) | 0 (0) | 6 (1.6) | 0 (0) | 0 (0) | 0 (0) | p=.907 |
|  | 6-10 years | 10 (8.6) | 0 (0) | 6 (9.4) | 4 (12.1) | 0 (0) | 0 (0) |  |
|  | 11-20 years | 38 (32.8) | 3 (42.9) | 21 (32.8) | 10 (30.3) | 3 (33.3) | 1 (33.3) |  |
|  | 21-30 years | 40 (34.5) | 3 (42.9) | 19 (29.7) | 12 (36.4) | 5 (55.6) | 1 (33.3) |  |
|  | More than 30 years | 27 (23.3) | 1 (14.3) | 17 (26.6) | 7 (21.2) | 1 (11.1) | 1 (33.3) |  |
|  | Not specified | 0 (0) | 0 (0) | 0 (0) | 0 (0) | 0 (0) | 0 (0) |  |
| Federal state |  |  |  |  |  |  |  |  |
|  | Baden-Wuerttemberg | 21 (18.1) | 1 (14.3) | 14 (21.9) | 6 (18.2) | 0 (0) | 0 (0) | V=0.354 |
|  | Bavaria | 12 (10.3) | 2 (28.6) | 4 (6.3) | 4 (12.1) | 1 (11.1) | 1 (33.3) | p=.685 |
|  | Berlin | 7 (6) | 0 (0) | 3 (4.7) | 2 (6.1) | 1 (11.1) | 1 (33.3) |  |
|  | Brandenburg | 1 (0.9) | 0 (0) | 1 (1.6) | 0 (0) | 0 (0) | 0 (0) |  |
|  | Bremen | 1 (0.90) | 0 (0) | 0 (0) | 1 (3) | 0 (0) | 0 (0) |  |
|  | Hamburg | 4 (3.40) | 1 (14.3) | 2 (3.1) | 1 (3) | 0 (0) | 0 (0) |  |
|  | Hesse | 9 (7.8) | 0 (0) | 6 (9.4) | 3 (9.1) | 0 (0) | 0 (0) |  |
|  | Mecklenburg-Western Pomerania | 3 (2.6) | 0 (0) | 3 (4.7) | 0 (0) | 0 (0) | 0 (0) |  |
|  | Lower Saxony | 10 (8.6) | 2 (28.6) | 5 (7.8) | 3 (9.1) | 0 (0) | 0 (0) |  |
|  | North Rhine-Westphalia | 16 (13.8) | 0 (0) | 7 (10.9) | 7 (21.2) | 2 (22.2) | 0 (0) |  |
|  | Rhineland-Palatinate | 6 (5.2) | 0 (0) | 3 (4.7) | 2 (6.1) | 1 (11.1) | 0 (0) |  |
|  | Saarland | 2 (1.7) | 0 (0) | 2 (3.1) | 0 (0) | 0 (0) | 0 (0) |  |
|  | Saxony | 14 (12.1) | 1 (14.3) | 8 (12.5) | 3 (9.1) | 1 (11.1) | 1 (33.3) |  |
|  | Saxony-Anhalt | 3 (2.6) | 0 (0) | 3 (4.7) | 0 (0) | 0 (0) | 0 (0) |  |
|  | Schleswig-Holstein | 3 (2.6) | 0 (0) | 1 (1.6) | 0 (0) | 2 (22.2) | 0 (0.0) |  |
|  | Thuringia | 2 (1.7) | 0 (0) | 1 (1.6) | 1 (3) | 0 (0) | 0 (0) |  |
|  | Not specified | 2 (1.7) | 0 (0) | 1 (1.6) | 1 (3) | 0 (0) | 0 (0) |  |
| Activity in a municipality/city with |  |  |  |  |  |  |  |  |
|  | Less than 5,000 inhabitants | 5 (4.3) | 0 (0.) | 3 (4.7) | 2 (6.1) | 0 (0) | 0 (0) | V=0.127 |
|  | 5,000 to 20,000 inhabitants | 28 (24.1) | 0 (0) | 17 (26.6) | 9 (27.3) | 2 (22.2) | 0 (0) | p=.961 |
|  | 20,001 to 100,000 inhabitants | 26 (22.4) | 3 (42.9) | 15 (23.4) | 5 (15.2) | 2 (22.2) | 1 (33.3) | Fisher exact=.954 |
|  | 100,001 to 500,000 inhabitants | 25 (21.6) | 2 (28.6) | 14 (21.9) | 6 (18.2) | 2 (22.2) | 1 (33.3) |  |
|  | More than 500,000 inhabitants | 32 (27.6) | 2 (28.6) | 15 (23.4) | 11 (33.3) | 3 (33.3) | 1 (33.3) |  |
|  | Not specified | 0 (0) | 0 (0) | 0 (0) | 0 (0) | 0 (0) | 0 (0) |  |
| Working model |  |  |  |  |  |  |  |  |
|  | Individual practice (without other colleagues) | 12 (10.3) | 0 (0) | 4 (6.3) | 6 (18.2) | 2 (22.2) | 0 (0) | V=0.164 |
|  | Individual practice (with employed doctors) | 22 (19) | 2 (28.6) | 12 (18.8) | 5 (15.2) | 2 (22.2) | 1 (33.3) | p=.974 |
|  | Group practice | 46 (39.7) | 4 (57.1) | 26 (40.6) | 12 (36.4) | 3 (33.3) | 1 (33.3) | Fisher exact=.964 |
|  | Medical care center | 18 (15.5) | 1 (14.3) | 10 (15.6) | 5 (15.2) | 1 (11.1) | 1 (33.3) |  |
|  | Hospital | 14 (12.1) | 0 (0) | 8 (12.5) | 5 (15.2) | 1 (11.1) | 0 (0) |  |
|  | Other | 3 (2.6) | 0 (0) | 3 (4.7) | 0 (0) | 0 (0) | 0 (0) |  |
|  | Not specified | 0 (0) | 0 (0) | 0 (0) | 0 (0) | 0 (0) | 0 (0) |  |
| Patients treated per quarter |  |  |  |  |  |  |  |  |
|  | Less than 500 | 7 (6) | 0 (0) | 5 (7.8) | 2 (6.1) | 0 (0) | 0 (0) | ρ=0.086 |
|  | 500 to 750 | 12 (10.3) | 0 (0) | 7 (10.9) | 4 (12.1) | 1 (11.1) | 0 (0) | p=.361 |
|  | 751 to 1000 | 25 (21.6) | 1 (14.3) | 18 (28.1) | 5 (15.2) | 0 (0) | 1 (33.3) |  |
|  | 1001 to 1500 | 29 (25) | 2 (28.6) | 16 (25) | 7 (21.2) | 3 (33.3) | 1 (33.3) |  |
|  | 1501 to 2000 | 19 (16.4) | 0 (0) | 9 (14.1) | 6 (18.2) | 3 (33.3) | 1 (33.3) |  |
|  | More than 2000 | 22 (19) | 4 (57.1) | 8 (12.5) | 8 (24.2) | 2 (22.2) | 0 (0) |  |
|  | Not specified | 2 (1.7) | 0 (0) | 1 (1.6) | 1 (3) | 0 (0) | 0 (0) |  |
| Ever used a health app as a patient |  |  |  |  |  |  |  |  |
|  | Yes | 55 (47) | 4 (57.1) | 28 (43.8) | 15 (44.1) | 7 (77.8) | 1 (33.3) | V=0.192 |
|  | No | 62 (53) | 3 (42.9) | 36 (56.3) | 19 (55.9) | 2 (22.2) | 2 (66.7) | p=.364 |
|  |  |  |  |  |  |  |  | Fisher exact=.394 |
| Ever used a DiHA as a patient |  |  |  |  |  |  |  |  |
|  | Yes | 15 (12.8) | 0 (0) | 5 (7.8) | 7 (20.6) | 3 (33.3) | 0 (0) | V=0.264 |
|  | No | 102 (87.2) | 7 (100) | 59 (92.2) | 27 (79.4) | 6 (66.7) | 3 (100) | p=.087 |
|  |  |  |  |  |  |  |  | Fisher exact=.098 |
| Ever used a DiHA manufacturer access |  |  |  |  |  |  |  |  |
|  | Yes | 72 (61.5) | 3 (42.9) | 38 (59.4) | 20 (58.8) | 9 (100) | 2 (66.7) | V=0.243 |
|  | No | 45 (38.5) | 4 (57.1) | 26 (40.6) | 34 (100) | 9 (100) | 3 (100) | p=.140 |
|  |  |  |  |  |  |  |  | Fisher exact=.134 |

^A^ from the indication area hormones and metabolism

ρ=Spearman’s rank correlation coefficient| p=p-value | V=Cramér’s V

The descriptive statistics are to be interpreted within the variable “Prescription frequency”.
