# Supplementary material for: Prescribing Experiences, Potentials, and Challenges of Digital Health Applications in the Field of Hormones and Metabolism: Cross-Sectional Survey Study of Health Care Providers in Germany
Source: JMIR Form Res. 2025 Dec 31;9:e77792. doi: 10.2196/77792 (PMC12805319; doi:10.2196/77792)
Supplement: Multimedia Appendix 8 [file formative_v9i1e77792_app8.docx]

Multimedia Appendix 8: Assessment of potential (N=325) and actual (N=126) healthcare effects, n, (%)

| The use of DiHA from the indication area of hormones and metabolism … | Do not agree at all | Do not agree | Undecided | Agree | Agree completely | I do not know |
| --- | --- | --- | --- | --- | --- | --- |
|  |  |  |  |  |  |  |
| ... could prolong survival. | 27 (8.3) | 44 (13.5) | 108 (33.2) | 100 (30.8) | 17 (5.2) | 29 (8.9) |
| ... has prolonged survival. | 20 (15.9) | 29 (23) | 39 (31) | 14 (11.1) | 1 (0.8) | 23 (18.3) |
| ... could reduce discomfort and complications. | 17 (5.2) | 16 (4.9) | 56 (17) | 185 (56.9) | 38 (11.7) | 13 (4) |
| ... has reduced the complaints and complications. | 13 (10.3) | 13 (10.3) | 33 (26.2) | 51 (40.5) | 5 (4) | 11 (8.7) |
| ... could improve the quality of life. | 15 (4.6) | 14 (4.3) | 49 (15.1) | 178 (54.8) | 59 (18.2) | 10 (3.1) |
| ... has increased the quality of life. | 9 (7.1) | 10 (7.9) | 27 (21.4) | 60 (47.6) | 10 (7.9) | 10 (7.9) |
| ... could increase the alignment of treatment with guidelines and recognized standards. | 13 (4) | 23 (7.1) | 68 (20.9) | 173 (53.2) | 34 (10.5) | 14 (4.3) |
| ... has increased the alignment of treatment with guidelines and recognized standards. | 10 (7.9) | 22 (17.5) | 40 (31.7) | 38 (30.2) | 5 (4) | 11 (8.7) |
| ... could increase adherence to treatment. | 14 (4.3) | 14 (4.3) | 48 (14.8) | 197 (60.6) | 43 (13.2) | 9 (2.8) |
| ... has increased adherence to treatment. | 7 (5.6) | 13 (10.3) | 33 (26.2) | 59 (46.8) | 6 (4.8) | 8 (6.3) |
| ... could improve access to hard-to-reach patient groups. | 24 (7.4) | 59 (18.2) | 82 (25.2) | 120 (36.9) | 26 (8) | 14 (4.3) |
| ... has improved access to hard-to-reach patient groups. | 13 (10.3) | 30 (23.8) | 32 (25.4) | 33 (26.2) | 8 (6.3) | 10 (7.9) |
| ... could increase patient safety. | 21 (6.5) | 35 (10.8) | 85 (26.2) | 148 (45.5) | 22 (6.8) | 14 (4.3) |
| ... has increased patient safety. | 10 (7.9) | 17 (13.5) | 39 (31) | 45 (35.7) | 2 (1.6) | 13 (10.3) |
| ... could increase health literacy. | 14 (4.3) | 14 (4.3) | 40 (12.3) | 188 (57.8) | 58 (17.8) | 11 (3.4) |
| ... has increased health literacy. | 6 (4.8) | 6 (4.8) | 29 (23) | 68 (54) | 10 (7.9) | 7 (5.6) |
| ... could increase patient sovereignty. | 12 (3.7) | 15 (4.6) | 59 (18.2) | 171 (52.6) | 56 (17.2) | 12 (3.7) |
| ... has increased patient sovereignty. | 4 (3.2) | 10 (7.9) | 28 (22.2) | 66 (52.4) | 9 (7.1) | 9 (7.1) |
| ... could improve disease management. | 14 (4.3) | 21 (6.5) | 53 (16.4) | 178 (54.9) | 48 (14.8) | 10 (3.1) |
| … has improved disease management. | 7 (5.6) | 10 (7.9) | 33 (26.2) | 62 (49.2) | 7 (5.6) | 7 (5.6) |
| ... could improve the involvement of relatives in the care process. | 17 (5.2) | 52 (16) | 94 (28.9) | 120 (36.9) | 24 (7.4) | 18 (5.5) |
| ... has improved the involvement of relatives in the care process. | 12 (9.5) | 26 (20.6) | 47 (37.3) | 22 (17.5) | 2 (1.6) | 17 (13.5) |
| ... could reduce the HbA1c value. | 14 (4.3) | 19 (5.8) | 66 (20.3) | 174 (53.5) | 33 (10.2) | 19 (5.8) |
| ... has reduced the HbA1c value. | 9 (7.1) | 22 (17.5) | 30 (23.8) | 46 (36.5) | 6 (4.8) | 13 (10.3) |
| ... could reduce the weight. | 13 (4) | 21 (6.5) | 65 (19.7) | 179 (55.1) | 36 (11.1) | 12 (3.7) |
| ... has reduced the weight. | 7 (5.6) | 12 (9.5) | 31 (24.6) | 60 (47.6) | 7 (5.6) | 9 (7.1) |
| ... could improve self-management. | 14 (4.3) | 8 (2.5) | 30 (9.3) | 205 (63.3) | 62 (19.1) | 5 (1.5) |
| ... has improved self-management. | 5 (4) | 10 (7.9) | 25 (19.8) | 65 (51.6) | 13 (10.3) | 8 (6.3) |
