# Supplementary material for: Prescribing Experiences, Potentials, and Challenges of Digital Health Applications in the Field of Hormones and Metabolism: Cross-Sectional Survey Study of Health Care Providers in Germany
Source: JMIR Form Res. 2025 Dec 31;9:e77792. doi: 10.2196/77792 (PMC12805319; doi:10.2196/77792)
Supplement: Multimedia Appendix 9 [file formative_v9i1e77792_app9.docx]

Multimedia Appendix 9: Correlation between healthcare effects and prescription experience, prescription frequency and prescription intention

| The use of DiHA from the indication area of hormones and metabolism … | General DiHA prescription | | DiHA prescription from the indication area hormones and metabolism | | Prescription frequency | | Prescription intention | |
| --- | --- | --- | --- | --- | --- | --- | --- | --- |
|  |  | |  | |  | |  | |
|  | Cramér's V | p-value | Cramér's V | p-value | ρ | p-value | ρ | p-value |
|  |  |  |  |  |  |  |  |  |
| ... could prolong survival. | 0.183 | .055 | 0.124 | .782 | 0.281 | <.001 | 0.162 | .003 |
| ... has prolonged survival. | n.a. | n.a. | n.a. | n.a. | n.a. | n.a. | 0.173 | .053 |
| ... could reduce discomfort and complications. | 0.249 | .001 | 0.249 | .080 | 0.212 | .013 | 0.266 | <.001 |
| ... has reduced the complaints and complications. | n.a. | n.a. | n.a. | n.a. | n.a. | n.a. | 0.210 | .018 |
| ... could improve the quality of life. | 0.240 | .002 | 0.236 | .114 | 0.233 | .006 | 0.336 | <.001 |
| ... has increased the quality of life. | n.a. | n.a. | n.a. | n.a. | n.a. | n.a. | 0.723 | .002 |
| ... could increase the alignment of treatment with guidelines and recognized standards. | 0.228 | .005 | 0.167 | .491 | 0.297 | <.001 | 0.286 | <.001 |
| ... has increased the alignment of treatment with guidelines and recognized standards. | n.a. | n.a. | n.a. | n.a. | n.a. | n.a. | 0.302 | <.001 |
| ... could increase adherence to treatment. | 0.190 | .039 | 0.096 | .916 | 0.212 | .013 | 0.251 | <.001 |
| ... has increased adherence to treatment. | n.a. | n.a. | n.a. | n.a. | n.a. | n.a. | 0.345 | <.001 |
| ... could improve access to hard-to-reach patient groups. | 0.157 | .157 | 0.253 | .071 | 0.188 | .029 | 0.122 | .028 |
| ... has improved access to hard-to-reach patient groups. | n.a. | n.a. | n.a. | n.a. | n.a. | n.a. | 0.153 | .087 |
| ... could increase patient safety. | 0.184 | .053 | 0.052 | .995 | 0.190 | .027 | 0.211 | <.001 |
| ... has increased patient safety. | n.a. | n.a. | n.a. | n.a. | n.a. | n.a. | 0.249 | .005 |
| ... could increase health literacy. | 0.184 | .051 | 0.139 | .686 | 0.202 | .019 | 0.239 | <.001 |
| ... has increased health literacy. | n.a. | n.a. | n.a. | n.a. | n.a. | n.a. | 0.259 | .003 |
| ... could increase patient sovereignty. | 0.195 | .031 | 0.133 | .728 | 0.229 | .007 | 0.267 | <.001 |
| ... has increased patient sovereignty. | n.a. | n.a. | n.a. | n.a. | n.a. | n.a. | 0.334 | <.001 |
| ... could improve disease management. | 0.221 | .008 | 0.116 | .833 | 0.239 | .005 | 0.330 | <.001 |
| … has improved disease management. | n.a. | n.a. |  |  | n.a. | n.a. | 0.295 | <.001 |
| ... could improve the involvement of relatives in the care process. | 0.227 | .005 | 0.145 | .646 | 0.070 | .419 | 0.172 | .002 |
| ... has improved the involvement of relatives in the care process. | n.a. | n.a. | n.a. | n.a. | n.a. | n.a. | 0.208 | .020 |
| ... could reduce the HbA1c value. | 0.225 | .006 | 0.151 | .604 | 0.217 | .011 | 0.202 | <.001 |
| ... has reduced the HbA1c value. | n.a. | n.a. | n.a. | n.a. | n.a. | n.a. | 0.298 | <.001 |
| ... could reduce the weight. | 0.227 | .005 | 0.205 | .247 | 0.245 | .004 | 0.272 | <.001 |
| ... has reduced the weight. | n.a. | n.a. | n.a. | n.a. | n.a. | n.a. | 0.305 | <.001 |
| ... could improve self-management. | 0.173 | .086 | 0.256 | .066 | 0.110 | .204 | 0.351 | <.001 |
| ... has improved self-management. | n.a. | n.a. | n.a. | n.a. | n.a. | n.a. | 0.260 | .003 |

n.a=not applicable
