# Supplementary material for: Prescribing Experiences, Potentials, and Challenges of Digital Health Applications in the Field of Hormones and Metabolism: Cross-Sectional Survey Study of Health Care Providers in Germany
Source: JMIR Form Res. 2025 Dec 31;9:e77792. doi: 10.2196/77792 (PMC12805319; doi:10.2196/77792)
Supplement: Multimedia Appendix 10 [file formative_v9i1e77792_app10.docx]

Multimedia Appendix 10: Assessment of barriers (N=298) for DiHA prescription, n (%)

| In your opinion, what are the biggest problems and barriers to prescribing DiHA from the indication area of hormones and metabolism? | Do not agree at all | Do not agree | Undecided | Agree | Agree completely | I do not know |
| --- | --- | --- | --- | --- | --- | --- |
|  |  |  |  |  |  |  |
| Lack of or insufficient evidence of benefit for patients | 3 (1) | 60 (20.1) | 78 (26.2) | 109 (36.6) | 33 (11.1) | 15 (5) |
| Application too complicated for patients | 1 (0.3) | 76 (25.5) | 84 (28.2) | 102 (34.2) | 18 (6) | 17 (5.7) |
| Lack of technical support from the manufacturer | 3 (1) | 68 (22.8) | 100 (33.6) | 75 (25.2) | 19 (6.4) | 33 (11.1) |
| Lack of patient motivation | 4 (1.3) | 44 (14.8) | 67 (22.5) | 122 (40.9) | 49 (16.4) | 12 (4) |
| Uncertainty regarding the protection of privacy and the security of patients' personal (health) data | 10 (3.4) | 83 (27.9) | 60 (20.1) | 103 (34.6) | 27 (9.1) | 15 (5) |
| Physician-patient relationship becomes more impersonal | 16 (5.4) | 136 (45.6) | 50 (16.8) | 62 (20.8) | 29 (9.7) | 5 (1.7) |
| Lack of digital literacy among patients | 6 (2) | 56 (18.8) | 67 (22.5) | 128 (43) | 36 (12.1) | 5 (1.7) |
| Insufficient adaptation of the DiHA to the individual needs of patients | 0 (0) | 41 (13.8) | 96 (32.2) | 105 (35.2) | 33 (11.1) | 23 (7) |
| Poor integration or compatibility with existing practice software and tools | 3 (1) | 18 (6) | 54 (18.1) | 114 (38.3) | 81 (27.2) | 28 (9.4) |
| Insufficient reimbursement of ancillary medical services, e.g. monitoring patient data and responding to queries | 3 (1) | 19 (6.4) | 38 (12.8) | 135 (45.3) | 84 (28.2) | 19 (6.4) |
