# Supplementary material for: Prescribing Experiences, Potentials, and Challenges of Digital Health Applications in the Field of Hormones and Metabolism: Cross-Sectional Survey Study of Health Care Providers in Germany
Source: JMIR Form Res. 2025 Dec 31;9:e77792. doi: 10.2196/77792 (PMC12805319; doi:10.2196/77792)
Supplement: Multimedia Appendix 11 [file formative_v9i1e77792_app11.docx]

Multimedia Appendix 11: Correlation between barriers and prescription experience, prescription frequency and prescription intention

| In your opinion, what are the biggest problems and barriers to prescribing DiHA from the indication area of hormones and metabolism? | General DiHA prescription | | DiHA prescription from the indication area hormones and metabolism | | Prescription frequency | | Prescription intention | |
| --- | --- | --- | --- | --- | --- | --- | --- | --- |
|  |  | |  | |  | |  | |
|  | Cramér's V | p-value | Cramér's V | p-value | ρ | p-Wert | ρ | p-value |
|  |  |  |  |  |  |  |  |  |
| Lack of or insufficient evidence of benefit for patients | 0.247 | .003 | 0.188 | .423 | -0.138 | .133 | -0.276 | <.001 |
| Application too complicated for patients | 0.167 | .141 | 0.214 | .267 | 0.056 | .545 | -0.130 | .025 |
| Lack of technical support from the manufacturer | 0.195 | .046 | 0.238 | .159 | -0.031 | .735 | -0.209 | <.001 |
| Lack of patient motivation | 0.189 | .060 | 0.232 | .186 | 0.039 | .674 | -0.007 | .906 |
| Uncertainty regarding the protection of privacy and the security of patients' personal (health) data | 0.180 | .085 | 0.108 | .899 | -0.081 | .381 | -0.089 | .123 |
| Physician-patient relationship becomes more impersonal | 0.104 | .670 | 0.147 | .698 | -0.070 | .447 | -0.221 | <.001 |
| Lack of digital literacy among patients | 0.132 | .396 | 0.139 | .745 | 0.015 | .874 | 0.010 | .866 |
| Insufficient adaptation of the DiHA to the individual needs of patients | 0.133 | .257 | 0.073 | .945 | -0.382 | <.001 | -0.157 | .007 |
| Poor integration or compatibility with existing practice software and tools | 0.241 | .004 | 0.159 | .619 | -0.118 | .199 | -0.149 | .010 |
| Insufficient reimbursement of ancillary medical services, e.g. monitoring patient data and responding to queries | 0.223 | .011 | 0.143 | .721 | -0.080 | .386 | -0.116 | .045 |
